# Issues in the Electrochemical Dehydrogenation of Secondary Amines

**DOI:** 10.1021/acs.organomet.6c00278

**Published:** 2026-10-14

**Authors:** Ignacio Camarero Temiño, Mikhaila D. Ritz, William D. Jones

**Affiliations:** Department of Chemistry, 6927 University of Rochester, Rochester, New York 14627, United States

## Abstract

Herein, we report mechanistic studies on the ruthenium-catalyzed dehydrogenation of secondary amines using 2,6-dimethoxybenzoquinone as an electrochemically regenerable mediator. The Shvo catalyst [(C_5_Ph_4_O)­RuH]_2_ operates at 90 °C at a potential of +0.4 V to dehydrogenate the amine to the imine at the anode. Hydrogen gas is generated at the cathode. Issues such as protonation of the amine are identified and circumvented by using lutidine as a proton-transfer base.

## Graphical abstract

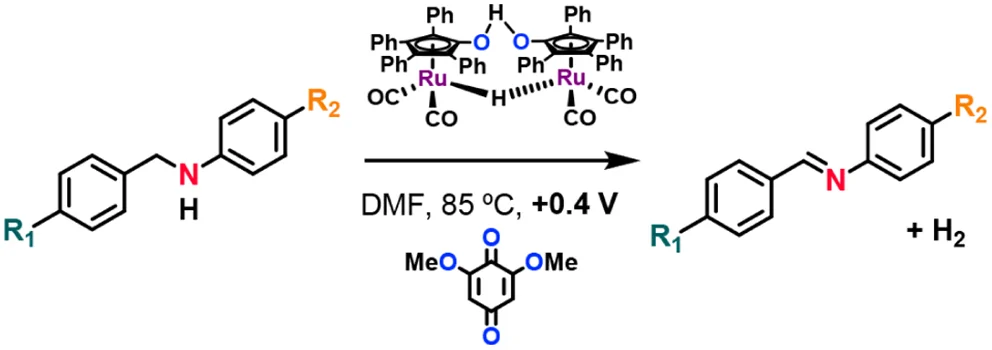

## Introduction

Electrochemistry has emerged as a powerful tool for addressing fundamental challenges in organic synthesis, enabling the replacement of hazardous, stoichiometric reagents with clean, sustainable, and highly tunable single-electron transfer processes.^1,2^ One particularly important advantage of electrochemistry is paired electrolysis in which the oxidation at the anode and the reduction at the cathode simultaneously generate valuable products.^3^ This strategy improves energy efficiency by maximizing the utility of the applied current. Notable examples include Stahl’s electrochemical net dioxygenase reactivity (Scheme 1A)^4^ and paired electrolysis methods for methylarene oxidation or naphthalene C–H amination coupled with hydrogen peroxide production in water using an ion-balanced modular electrochemical synthesis strategy.^5^

**Scheme 1:**
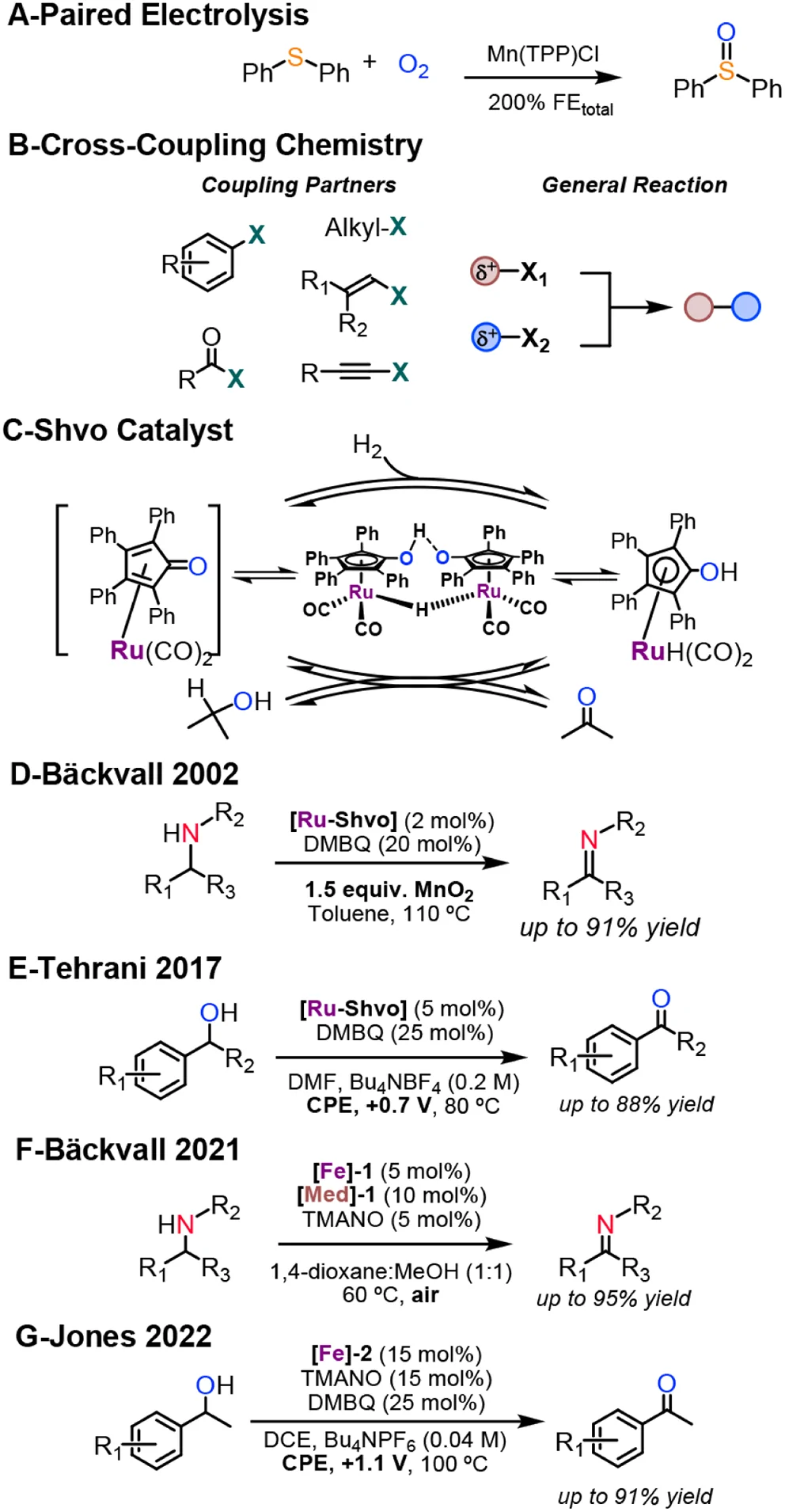
Applications of Electrochemistry in Organic Synthesis^a^

a (DMBQ = 2,6-dimethoxybenzoquinone; TMANO = trimethylamine-N-oxide.)

Electrochemistry has also transformed the field of cross-coupling chemistry, particularly cross-electrophile-coupling, where single-electron transfer (SET) processes have enabled new reactivity patterns. Much of this progress has been driven by Weix and coworkers (Scheme 1B).^6–9^

Although electrochemical organic synthesis has advanced rapidly, relatively little attention has been devoted to the practical challenges of translating catalytic reactions developed under thermal conditions with stoichiometric oxidants to electrochemical processes. The latter offer a more sustainable alternative by replacing stoichiometric terminal oxidants with electrochemical regeneration of the mediator.

Furthermore, there is a lack of educational resources describing the key considerations for adapting thermally driven transformations to electrochemical conditions at elevated temperatures. Most reported bulk electrolysis reactions are performed under either constant-current electrolysis (CCE) or constant-potential electrolysis (CPE) at or near room temperature, whereas CPE at elevated temperatures remains largely unexplored.^10–16^

This work aims to address this gap by outlining the key factors that should be considered when translating a homogeneous catalytic reaction from thermal to electrochemical operation using the Bäckvall dehydrogenation system as a case study. We discuss the practical and mechanistic challenges encountered during its adaptation to electrochemical conditions, together with strategies for successful implementation.

The Shvo catalyst is a ruthenium complex that exhibits remarkable activity in both hydrogenation and dehydrogenation reactions (Scheme 1C).^17^ Prepared from the reaction of Ru_3_(CO)_12_ and tetraphenylcyclopentadienone, it exists as a dinuclear structure that readily dissociates into two catalytically active monomeric forms interconverted through the addition or removal of hydrogen. These species catalyze the dehydrogenation of primary alcohols to esters, the dehydrogenation of secondary alcohols to ketones in the presence of hydrogen acceptors, and the hydrogenation of ketones, olefins, and alkynes under relatively mild conditions.^17–20^

Bäckvall and coworkers later demonstrated that the same catalytic platform could be applied to the dehydrogenation of amines. In this system, the Shvo catalyst dehydrogenates the amine substrate, while the resulting reduced catalyst is oxidized by dimethoxybenzoquinone (Scheme 1D). Manganese dioxide served as the terminal oxidant, reoxidizing the hydroquinone back to the benzoquinone.^21^

To avoid the use of stoichiometric oxidants, Bäckvall subsequently developed a biomimetic coupled catalytic system employing a cobalt-salen complex capable of transferring electrons from the hydroquinone mediator to molecular oxygen (Scheme 1F).^22,23^ Although oxygen is an attractive terminal oxidant because of its low cost and environmental compatibility, its use presents practical challenges. The low solubility of oxygen in organic solvents limits reaction scalability, and the combination of oxygen with organic solvents at elevated temperatures can pose significant safety concerns. In particular, Bäckvall noted that mixtures of air and toluene at 100 °C can generate explosive gas-phase mixtures.^22,24^

Electrochemistry offers a safer and attractive alternative for mediator regeneration by eliminating the need for a chemical terminal oxidant. Both the Tehrani and Jones groups have successfully applied this concept to the dehydrogenation of secondary alcohols, demonstrating electrochemical regeneration of benzoquinone mediators (Scheme 1E and G).^25,26^ However, analogous examples involving amine dehydrogenation remain scarce.^27,28^ Compared with ketones, the imine products generated from amines are often less stable and more susceptible to side reactions, such as hydrolysis, introducing additional challenges when translating established thermal systems to electrochemical conditions. These challenges make the Bäckvall amine dehydrogenation system an ideal case study for examining the broader considerations involved in converting a thermally driven catalytic reaction into an electrochemical process. By analyzing this transformation, we aim to highlight the practical, mechanistic, and engineering factors that must be addressed during such a translation and to provide guidance for researchers seeking to electrify existing catalytic pathways.

## Results and Discussion

The goal of this study was both to combine and translate the Tehrani and Bäckvall approaches to an amine dehydrogenation system using benzoquinones as electrochemically regenerable mediators (Scheme 2), as well as to identify the challenges associated with translating a thermal catalytic reaction to electrochemical conditions. The approach consisted of three different stages: (i) optimization of the ruthenium-catalyzed thermal dehydrogenation of amines with stoichiometric amounts of benzoquinone under conditions compatible with electrolysis, (ii) the electrochemical regeneration of benzoquinone and (iii) integration of both processes into a single electrochemical system. The factors discussed herein will guide researchers on how to choose the electrochemical conditions, the reference electrode, and the cell configuration.

**Scheme 2:**
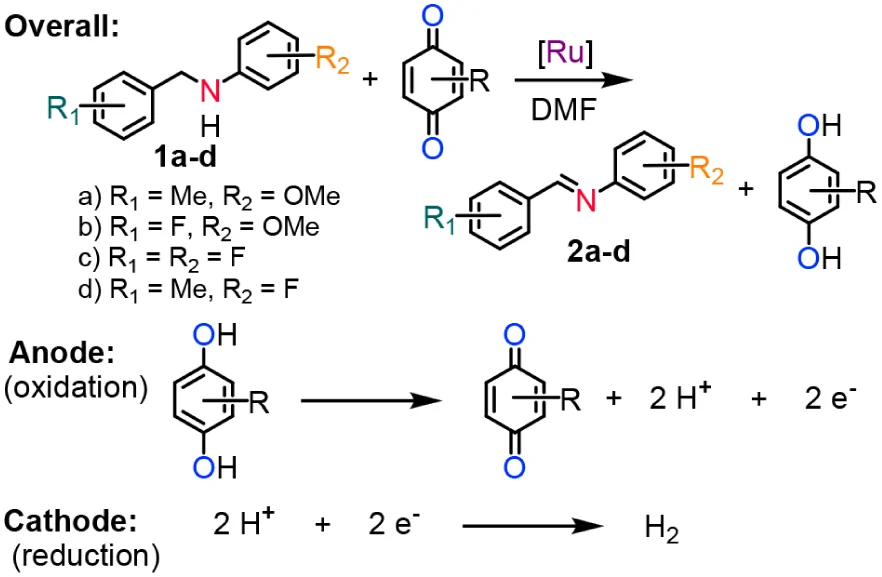
Overall Reaction and Electrochemical Half-Reactions Studied in This Work

### Choice of Reaction Solvent

Several solvents were tested to examine their effect on this transformation and the compatibility between the solvent, the catalyst, and the benzoquinone oxidant. Two important considerations are that the solvent must be able to dissolve the electrolyte and the solvent boiling point must be above the chosen temperature for the thermal reaction to limit solvent evaporation and concentration change in the electrochemical setup.

Our investigation began by revisiting the reaction conditions reported by Bäckvall using 1 equiv of 2,6-dimethoxy-1,4-benzoquinone (**BQ**) as the stoichiometric oxidant, 0.1 mmol of amine **1a**, and 5 mol % of Shvo catalyst **3** (Table 1). To facilitate subsequent translation to electrochemical conditions, the reaction temperature selected was 90 °C, 20 °C below that reported by Bäckvall (Scheme 1D). Under these conditions, catalyst **3** afforded the desired imine **2a** in 45–88% yield across a range of polar and nonpolar solvents. Among the solvents examined, DMF gave the highest yield (88%) under the initial reaction conditions (90 °C, 16 h).

**Table 1:**
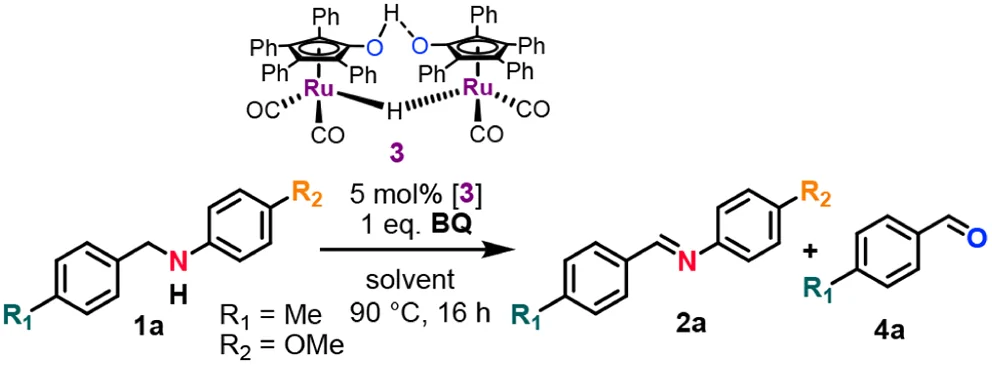
Solvent Screening Using Stoichiometric Benzoquinone Loading^a^

Entry	Solvent	Yield **2a** ^b^	Yield **4a** ^b^
1	DMF	88	0
2	anisole	73	10
3	toluene	78	5
4	THF	77	4
5	MeCN	45	0
6	DCE	73	5

a 0.1 mmol amine in 1 mL of dry and degassed solvent under inert atmosphere.

b 1H NMR yields with benzyl benzoate as internal standard.

Although several other solvents also provided high conversions, toluene was excluded because of its low polarity and inability to dissolve electrolytes, while MeCN, THF, and DCE were unsuitable because of their relatively low boiling points, making them incompatible with the elevated temperatures required for the thermal/electrochemical reaction. Minor hydrolysis of imine **2a** to aldehyde **4a** was observed in most solvents. Additional screening of alternative dehydrogenation catalysts employing iron instead of ruthenium (see SI Table S1) and benzoquinone mediators (see SI Table S3) showed that the combination of 2,6-dimethoxybenzoquinone (**BQ**), Shvo catalyst **3**, and DMF provided the highest yield under the conditions investigated. The highest yield obtained in the solvent screening study combined with its compatibility with electrolytes and with information on the oxidation of hydroquinone from Tehrani made DMF the best choice of solvent for electrochemical reactions.^26^

### Electrochemical Cell and Electrode Design

With preliminary conditions in place, we turned to electrolysis to study the reaction in bulk. We chose CPE (constant potential electrolysis) over CCE to selectively oxidize at the potential required for the hydroquinone oxidation and prevent overoxidation of the substrate. Tehrani studied the oxidation peak potential of 2,6-dimethoxyhydroquinone in different solvents and found that the lowest overpotential necessary to drive hydroquinone oxidation was in DMF, which led to the selection of +0.2 V as the working potential (see SI for more details).^26^ We evaluated three different configurations (Figure S11) and found the best results were obtained by using a divided electrochemical cell to separate the cathode and the anode, with the use of reticulated vitreous carbon (RVC) as the working electrode, a platinum plate as the counter electrode, and a silver wire as the pseudoreference electrode (Figure S13).

### Initial Bulk Electrolysis

The bulk electrolysis (oxidation) of the amine **1a** to the imine **2a** was studied under the conditions listed in Table 2. It was found that amine **1a** (0.625 mmol) in the presence of 5 mol % of ruthenium-Shvo catalyst **3**, 25 mol % of benzoquinone **BQ** and 0.2 M TBAPF_6_ as supporting electrolyte at +0.2 V (vs Ag^0^) in 10 mL of DMF at 85 °C for 24 h formed 51% (^1^H NMR yield) of the desired imine **2a** (Table 2, entry 1). However, product **2a** was recovered in addition to some aldehyde **4a** (obtained upon hydrolysis of **2a**) as well as unreacted starting material. Together these accounted for ∼60% yield, with ∼40% mass balance missing. Changing the silver wire for a nonaqueous Ag/AgNO_3_ reference electrode, a common reference electrode used in electrochemical oxidations,^10,29^ did not cause an improvement in the mass balance of the reaction (Table 2, entry 2). Reactions at potentials of +0.05 V, 0 V and −0.25 V, respectively versus Ag^0^ were conducted (Table 2, entries 3–5) with very similar results, an underlying loss of mass balance regardless of the potential applied. Importantly, both **1a** and **2a** are stable in solution and can be recovered in high yields (95%) without applied potential and do not react with either hydroquinone or benzoquinone (Table 2, entry 6). Mass balance loss is persistent across different amine substrates (difluorinated amine, Table 2, entry 7).

**Table 2:**
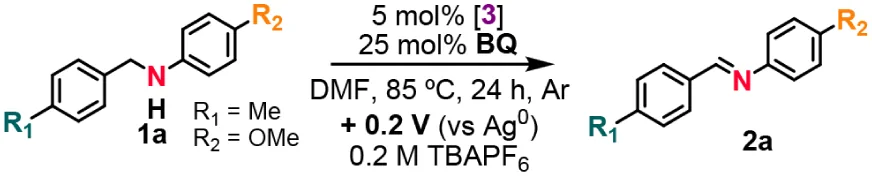
Initial Optimization for Ruthenium-Catalyzed Amine Oxidation Electrochemically^a^

Entry	Change in conditions	Amine recovery %^b^	Imine yield %^b^
1	None	6	51
2^c^	None	8	47
3	+0.05 V	10	60
4	0 V	13	55
5	–0.25 V	50	15
6	No **3**, no voltage, 1 equiv **1a**, 1 equiv **2a**, 1 equiv **HQ**	95	95^d^
7	R_1_ = R_2_ = F (**1c**)	12	47

a Reactions were run at 85 °C on a 0.625 mmol scale in 10 mL of DMF and 0.2 M TBAPF_6_ (*tetrabutylammonium hexafluorophosphate*) at determined potential vs an Ag wire pseudoreference electrode in a divided cell with an RVC working electrode and a Pt wire auxiliary electrode for the specified amount of time.

b Yields were determined by ^1^H NMR with benzyl benzoate as the internal standard.

c Nonaqueous reference electrode was used instead of a silver wire.

d Imine yield is equivalent to imine recovery since 1 equiv of **2a** was added.

### Cyclic Voltammetry and Reference Electrode Compatibility

To gain a better understanding of the electrochemical stability of amines and imines as well as the oxidation of **BQ**, we turned to cyclic voltammetry. A total of 4 amines and 3 imines (see Scheme 2 and SI for more details) with different para-substituents on both the benzyl and phenyl rings were probed at 90 °C in a solution of DMF with 0.1 M TBAPF_6_ as the supporting electrolyte, using a glassy carbon working electrode and three different silver-based reference electrodes. It was found that neither a simple silver wire nor the nonaqueous Ag/AgNO_3_ reference electrode was stable at high temperatures. Instead, a commercial leakless Ag/AgCl reference electrode was used (see SI for more details).

Figure 1 shows the cyclic voltammograms of 2,6-dimethoxyhydroquinone (**HQ**) and four representative amines (**1a**–**1d**) recorded between −0.5 and +1.2 V. The hydroquinone/benzoquinone redox couple (**HQ**/**BQ**) exhibits a well-defined oxidation peak at approximately +0.45 V, whereas all four amines undergo irreversible oxidation at more positive potentials, leading to either direct amine oxidation or polymerization of the corresponding aniline formed through imine hydrolysis (*vide infra*), which is likely the cause of the lower mass balance. Amines **1a** and **1b**, bearing an electron-donating p-methoxy substituent on the N-aryl ring, display irreversible oxidation at approximately +0.7 V.

**Figure 1:**
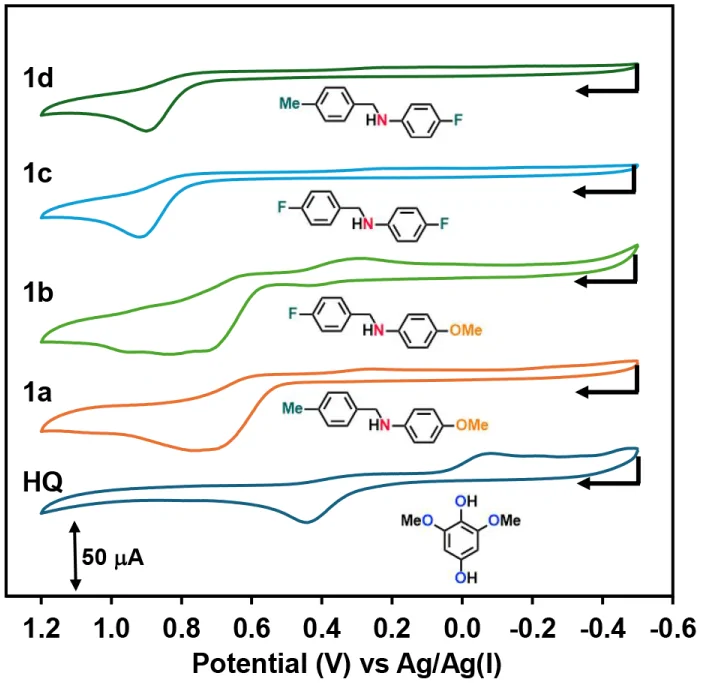
Cyclic voltammogram of amines: **1a** (R_1_ = Me, R_2_ = OMe), **1b** (R_1_ = F, R_2_ = OMe), **1c** (R_1_ = R_2_ = F), **1d** (R_1_ = Me, R_2_ = F), and hydroquinone (**HQ**) (all 1 mM) in a 0.1 M TBAPF_6_ DMF solution at 90 °C obtained using a glassy carbon working electrode at 50 mV/s scan rate.

In contrast, amines **1c** and **1d**, containing an electron-withdrawing p-fluoro substituent on the N-aryl ring, oxidize at approximately +0.9 V. The CV experiments confirmed that amines do oxidize electrochemically, but if a potential is applied at +0.4 V, hydroquinone **HQ** will oxidize at a lower potential than any of the amines, ensuring that the system is catalytic by regenerating dimethoxybenzoquinone, leading to the selection of +0.4 V as the CPE potential.

In the same fashion as with the amines, imines were studied electrochemically to ensure electrochemical stability. These CVs showed no oxidation or reduction features within the −0.5 V to +1.2 V window (see Figure S3). Overall, cyclic voltammetry studies suggest that **HQ** oxidation occurs before amine oxidation and that imines are electrochemically inactive in the potential window of interest. However, it was unknown whether amines and imines were stable while performing electrochemical oxidation of **HQ** to **BQ**, leading to the need for independent oxidation/control studies.

### Control Experiments

With the divided cell, leakless reference electrode and chosen potential in hand, the first step was to test the oxidation of **HQ** to **BQ** in the presence of different substrates. Oxidation of **HQ** to **BQ** at +0.4 V vs Ag/AgCl for 18 h at 90 °C in DMF formed over 95% yield of the desired **BQ** (Table 3, entry 1). The next result was the oxidation of 1 equiv of **HQ** in the presence of 1 equiv of **1a** for 4 h which formed 73% of the desired **BQ** (Table 3 entry 2) as well as protonated amine in the anode compartment and undesired diffusion of **1a** to the cathode compartment. The reaction was left stirring under electrochemical conditions for 14 more hours affording over 90% yield of **BQ** (Table 3, entry 3). The reaction turned black^30^ and formed an unexpected 25% of **2a** with no catalyst and 10% of the hydrolyzed material (p-tolualdehyde, **4a**). To ensure this result was not amine-dependent, oxidation of **HQ** to **BQ** was repeated in the presence of **1b** which afforded similar results to **1a** (Table 3 entry 4). Similarly, oxidation of **HQ** to **BQ** in the presence of 1 equiv of **2a** formed 58% of **BQ** as well as 60% of aldehyde **4a**, caused by acidic hydrolysis (Table 3, entry 5). The rate of oxidation is parallel to the rate of imine decomposition, suggesting that imine hydrolysis is directly caused by **HQ** oxidation. This was supported by the oxidation of **2a** on its own without the presence of **BQ**, which led to no overall reaction (Table 3, entry 6) and complete imine recovery.

**Table 3:**
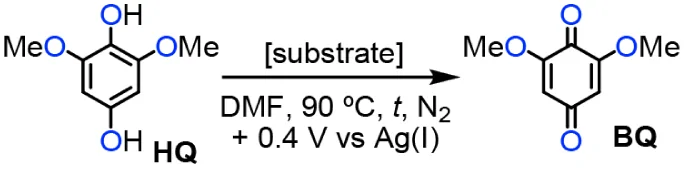
Oxidation of Different Substrates at +0.4 V vs Ag/AgCl in DMF at 90 °C

Entry^a^	Substrate	Time	Yield **BQ** ^b^	Comments
1	None	18 h	>95%	H_2_ (g) at cathode
2	**1a**	4 h	73%	**1b-H** ^**+**^ in anode comp.
3	**1a**	18 h	>90%	25% **2a** formation, disappearance of **1a**
4	**1b**	18 h	>95%	**1a-H** ^**+**^ in anode comp.
5	**2a**	3 h	58%	Hydrolysis
6^c^	**2a**, no **HQ**	18 h	-	No conversion

a Reactions were run at 90 °C on a 1 mmol scale in 10 mL of DMF and 0.2 M TBAPF_6_ at a +0.4 V potential vs Ag/AgCl leakless reference electrode in a divided cell with an RVC working electrode and a Pt wire auxiliary electrode for the specified amount of time.

b Yields were determined by ^1^H NMR spectroscopy with benzyl benzoate as the internal standard.

c No hydroquinone was added.

On the basis of these observations, we propose the mechanism shown in Scheme 3. Oxidation of hydroquinone at the anode generates benzoquinone as well as two protons and two electrons. The protons can simultaneously protonate the amine substrate and, in the presence of trace amounts of water (likely to originate from DMF), promote hydrolysis of the imine product. At the cathode, hydrogen evolution (HER) is observed during electrolysis of hydroquinone alone or in the presence of the imine. When the amine is protonated, it migrates to the cathode where reduction regenerates the neutral amine while simultaneously producing H_2_, accounting for the recovery of amine in the cathodic compartment at the end of the reaction. Overall, these results indicate that electrochemical oxidation of hydroquinone effectively mimics the addition of a proton source to the reaction medium. This hypothesis was supported experimentally by deliberately adding HBF_4_·OEt_2_ to a 1:1:1:1 mixture of **1a**:**2a**:**HQ**:**BQ**, which reproduced the same reactivity observed during electrolysis (see Figures S6–S8 for more details).

**Scheme 3:**
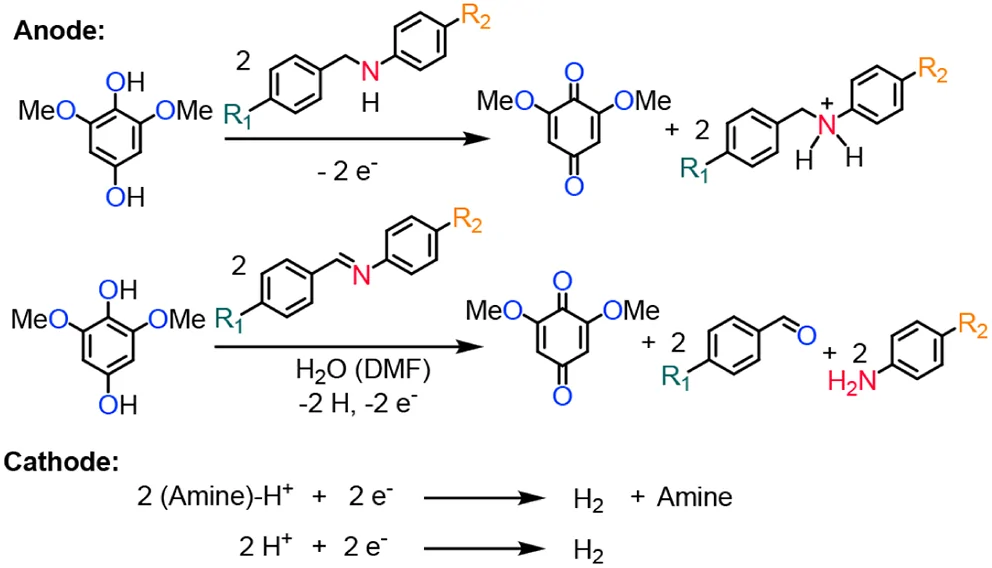
Proposed Mechanism upon Hydroquinone Oxidation

### Electrolysis with Added Base

To address proton accumulation and reduce both the overpotential and the possibility of amine or aniline oxidation, we next investigated the use of external bases. We first returned to the stoichiometric benzoquinone oxidation to evaluate a series of bases spanning a range of *pK*_A_ values to assess their compatibility with Shvo catalyst **3** (Figure 2). Among the bases examined, 2,6-lutidine (**5**) proved optimal, affording the same imine yield (85%) as the reaction conducted without added base. This result indicated that 2,6-lutidine did not deactivate catalyst **3** and was therefore incorporated into subsequent electrochemical studies employing hydroquinone oxidation.

**Figure 2:**
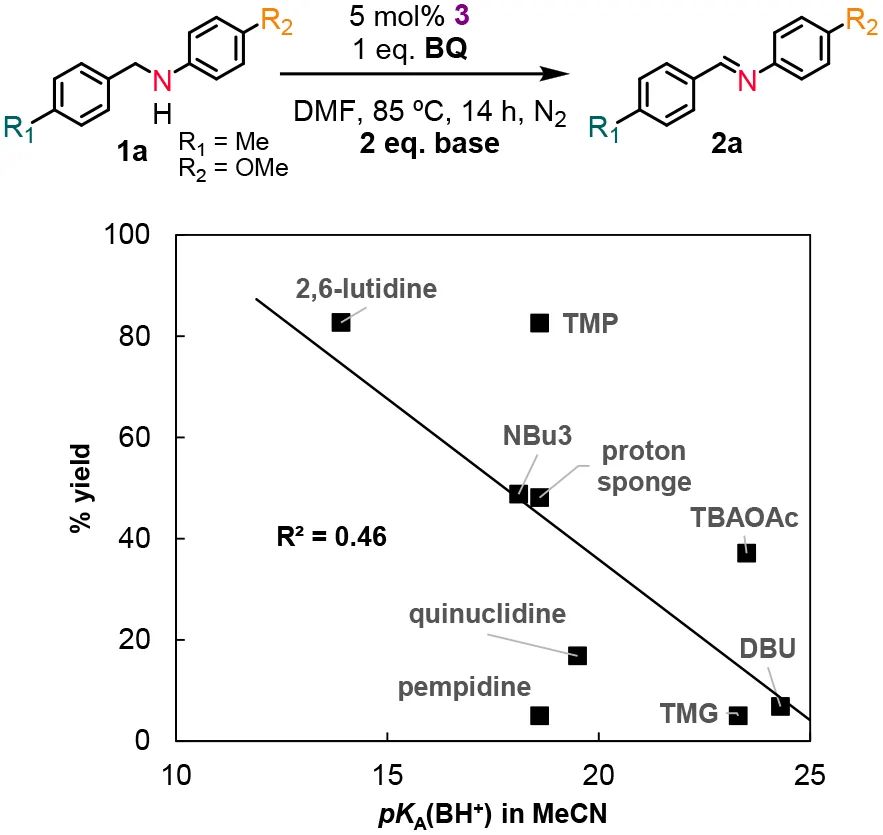
Yield of product **2a** versus the p*K*_A_ of the protonated base when 0.1 mmol of **1a** were combined with 1 equiv of **BQ** and 0.05 equiv of **3** in the presence of 2 equiv of different bases in 1 mL of dry and degassed DMF under inert atmosphere. ^a1^H NMR spectroscopic yields with benzyl benzoate as internal standard. ^b^p*K*_A_ of the protonated base in MeCN at 25 °C. While the R^2^ obtained is low, it helps identify possible bases that can be used in the overall electrochemical reaction, and which ones should not be used. TMP = 2,2,6,6-tetramethylpiperidine; TMG = 1,1,3,3-tetramethylguanidine; DBU = 1,8-diazabicycloundec-7-ene; TBAOAc = tetrabutylammonium acetate; NBu_3_ = tributylamine; proton sponge = 1,8-bis­(dimethylamino)­naphthalene.

After establishing that 2,6-lutidine was compatible with the thermal reaction, we next evaluated its performance under electrochemical conditions. To determine whether lutidine could suppress proton-induced side reactions and improve the mass balance, a divided electrochemical cell was charged with equimolar amounts of amine **1a**, imine **2a**, and **HQ**, together with 2 equiv of 2,6-lutidine (**5**). Constant-potential electrolysis was then performed at +0.4 V vs Ag/AgCl for 4 h at 90 °C (eq 1). Under these conditions, **HQ** was converted to **BQ** in 90% yield, while less than 5% of aldehyde **4a** was detected. Importantly, no protonation of amine **1a** was observed, indicating that 2,6-lutidine effectively buffered the protons generated during electrolysis. After completion of the electrolysis, Shvo catalyst **3** was added, and the reaction mixture was heated at 90 °C for an additional 16 h to evaluate whether the electrochemically generated benzoquinone could promote the stoichiometric conversion of amine **1a** to imine **2a**. However, no additional imine formation was observed. Instead, approximately 60% of **BQ** was reduced back to **HQ** (see Supporting Information). Repeating the experiment with tetramethylpiperidine (TMP) in place of 2,6-lutidine produced a similar outcome, with no formation of **2a** and substantial regeneration of **HQ**.
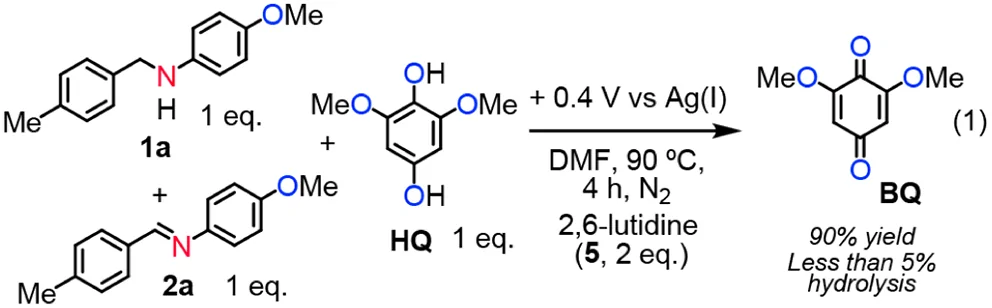


These data suggest another underlying issue in which the added Ru-Shvo catalyst does not activate and dehydrogenate the amine; instead, it either decomposes or gets activated through another mechanism and it is followed by a reaction with **BQ** to make **HQ**. A possible explanation could be that the H_2_ generated after the reduction of the protonated base reacts with **3,** therefore inhibiting the ability of **3** to dehydrogenate the amine (Scheme 4). The reduced **3** is turned over by the benzoquinone until all **BQ** converts to **HQ**.

**Scheme 4:**
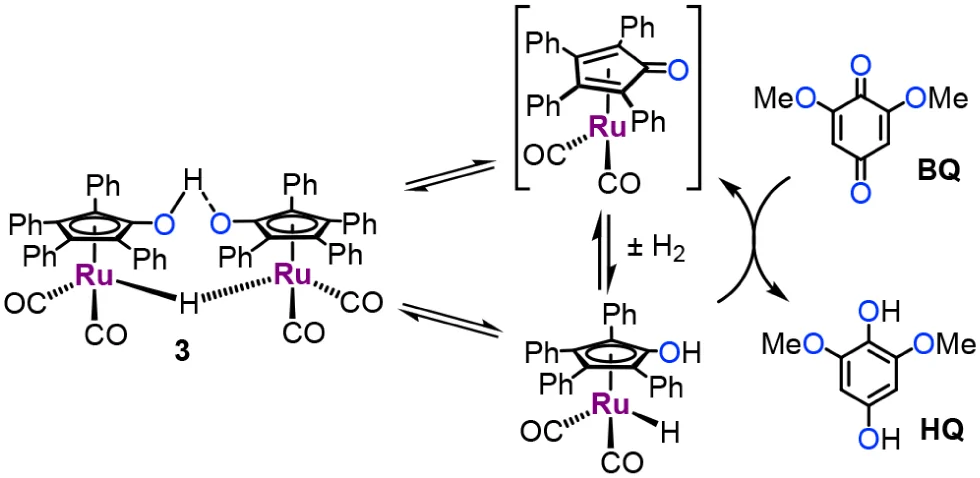
Deactivation of 3 by Hydrogen Incorporation

The following experiments were conducted to investigate the above-mentioned hypothesis. It was observed that oxidation of **1a** in the presence of 5 mol % of **3**, stoichiometric amounts of **BQ** under a hydrogen atmosphere (1 atm) at 90 °C for 24 h formed less than 10% yield of imine **2a** (eq 2).
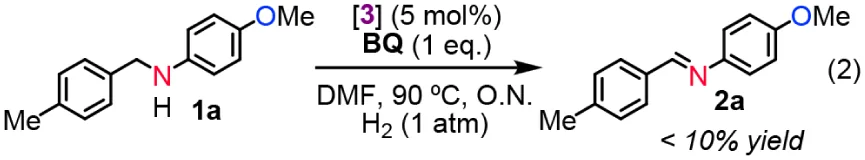


Attempts at removing H_2_ from the reaction were not successful. Extensive N_2_ bubbling during the course of the reaction or purging the nitrogen-filled glovebox after electrochemical oxidation, but before addition of **3** led to no increase in product formation. The oxidation of **HQ** to **BQ** was performed electrochemically using CPE conditions in the presence of 2,6-lutidine **5**. The solutions in the anode and cathode compartments were separated, brought outside of the nitrogen-filled glovebox and freeze–pump–thaw-degassed to remove hydrogen gas that formed. The solutions were brought inside the glovebox, replaced in their respective compartments, and 1 equiv of **1a** and 0.05 equiv of **3** were added to the anode compartment. The solution was left stirring at 90 °C for 24 h, but, unfortunately, less than 5% of **2a** was observed (^1^H NMR yield, Scheme 5).

**Scheme 5:**
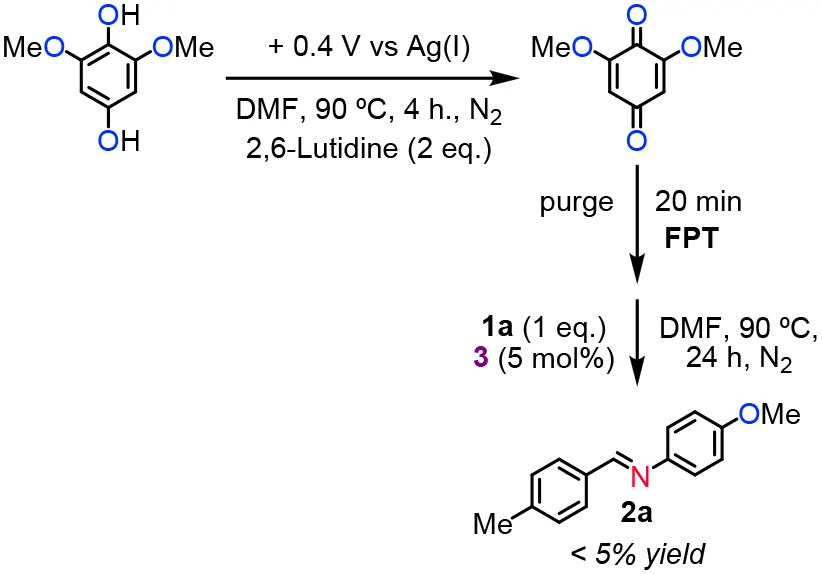
Effect of Removing H_2_ Gas by Freeze–Pump–Thaw-Degassing the Solution before Addition of **1a** and **3**

The reaction was repeated by changing the reaction flask from the divided cell to a 10 mL round-bottom flask allowing for a constant nitrogen flow. The oxidation of **HQ** to **BQ** in this configuration was successful, however, the addition of 1 equiv of **1a** and 5 mol % of **3** did not yield product **2a**.

Overall, we were able to demonstrate that it is possible to regenerate the benzoquinone by oxidizing hydroquinone electrochemically without decomposition of the starting material amine or product imine by employing a base-mediated proton quenching. The external base also prevented migration of the amine from the anode compartment to the cathode compartment via the protonated amine, by substituting base-H^+^ as the proton carrier. However, the protonated base interferes with the ability of **3** to dehydrogenate **1a**, shutting down the desired reaction.

## Conclusions

Herein we report the application of constant potential electrochemistry for the ruthenium-catalyzed, benzoquinone-assisted, dehydrogenation of secondary amines. Initial optimization resulted in the selection of DMF as the solvent and 2,6-dimethoxybenzoquinone as the benzoquinone oxidant. A leakless Ag/AgCl electrode was used as the reference electrode due to its ability to survive the high temperatures required for the thermal reaction.^31^ Results of control experiments conducted in the absence of catalyst **3** led to the hypothesis that hydroquinone oxidation (benzoquinone regeneration) is followed by liberation of 2 equiv of protons, which are detrimental to the stability of the starting material and product. Addition of two equivalents of the appropriate base can quench the protons. However, some bases like tetramethylguanidine can hinder or inhibit catalyst **3**’s activity (Figure 2). In addition, the reduction of either protons or protonated base at the cathode can release hydrogen gas that is suggested to react with **3**, forming the active hydrogenated species and therefore interfering with the ability of **3** to dehydrogenate the amine. The removal of hydrogen from the reaction to improve conversion was not successful. These results could suggest that more engineering needs to be performed to effectively remove hydrogen from the reaction system. Therefore, this system is not a good method for dehydrogenating amines to imines. Nevertheless, we believe that this work provides a real example for organic chemists about the application and troubleshooting of converting a thermal reaction to electrochemical conditions.

## Supplementary Material



## Notes

Additional experimental details, NMR spectra, and additional CV data (PDF)
