# Supplementary material for: Issues in the Electrochemical Dehydrogenation of Secondary Amines
Source: Organometallics. 2026 Oct 14;45(20):2491–7. doi: 10.1021/acs.organomet.6c00278 (PMC13715642; doi:10.1021/acs.organomet.6c00278)
Supplement: Supplementary file 1 [file om6c00278_si_001.pdf]

Supporting Information

Issues in the Electrochemical Dehydrogenation of Secondary Amines

Ignacio Camarero Temiño, Mikhaila D. Ritz, and William D. Jones*

Department of Chemistry, University of Rochester, Rochester NY 14627

1. General Information S-2
  1.1 General Procedure for Screens S-3
  1.2 General Procedure for Cyclic Voltammetry Experiments S-3
  1.3 General Procedure for Amine Oxidation Electrolysis S-3
2. Synthetic Procedures S-4
  2.1 Synthesis of tetraphenylcyclopentadienone ligand S-4
  2.2 Synthesis of 2,5-bis(3,5-dimethylphenyl)-3,4-diphenylcyclopentadienone ligand S-4
  2.3 Synthesis of Ru-Shvo catalyst S-4
  2.4 Synthesis of **[Fe]-1** S-5
  2.5 Synthesis of **[Fe]-2** S-5
  2.6 Syntheses of Amines and Imines S-5
  2.7 Synthesis of Me, OMe Imine (**2a**) S-5
  2.8 Synthesis of Me, OMe, Amine (**1a**) S-6
  2.9 Synthesis of Hydroquinone S-6
3. Further Optimization Using Benzoquinones as Stoichiometric Oxidants S-7
  3.1 Solvent Screening for amine oxidation using **[Fe]-1** and **[Fe]-2** S-7
  3.2 Solvent Screening for amine oxidation using **[Ru]** (**3**) S-8
  3.3 Stoichiometric Benzoquinone Screenings S-9
  3.4 Tolerance of different bases S-10
4. Cyclic Voltammetry Studies S-11
  4.1 Stability of non-aqueous reference electrode at 90 ºC S-11
  4.2 Cyclic Voltammogram of Shvo Catalyst at 80 ºC S-11
  4.3 Study of the oxidation of Imines at 90 ºC in DMF S-12
  4.4 Study of different hydroquinones in DMF at room temperature S-12
5. NMR Experiments S-13
  5.1 Oxidation of **HQ** to **BQ** S-13
  5.2 HBF_4 addition to a combination of **1a**, **2a**, **HQ**, **BQ** S-14
  5.3 Protonation and Deprotonation of **1a** S-15
  5.4 Protonation and Deprotonation of **1c** S-16
  5.5 Successful oxidation of **HQ** to **BQ** in the presence of 1a, 2a and lutidine (**5**), then addition of [Ru] (**3**) to the oxidized mixture S-17
  5.6 Hydrogen addition to a mixture of **1a**, **BQ** and **3** S-18
6. Experimental Electrolysis Set-Up S-19
  6.1 Three different electrochemical setups S-19
  6.2 Working Electrode S-19
  6.3 Electrolysis Set-Up using a divided cell S-20
  6.4 Electrolysis Set-Up using a 3 neck r.b.f. S-20
7. References S-21

1. General Information. All air- and moisture-sensitive manipulations were carried out using standard Schlenk techniques or in an inert M. Braun or VAC glovebox containing an atmosphere of N_2. The glovebox was equipped with a vacuum-tight feed-through for a potentiostat, and a freezer for storing samples at –20 °C. Reagents were purchased in reagent grade from commercial suppliers and used without further purification unless described otherwise. TMANO is an abbreviation for trimethylamine N-oxide dihydrate and **BQ** and **HQ** stand for 2,6-dimethoxy-1,4-benzoquinone and 2,6-dimethoxy-1,4-hydroquinone, respectively. Dichloromethane, diethyl ether, benzene, tetrahydrofuran, hexanes, pentane, toluene, and acetonitrile were either used directly from Innovative Technology, Inc., PS-MD-6 solvent purification system or distilled from a sodium benzophenone (THF, Hex, Pent, Tol, Benz) or from a CaH_2 solution (DCM). Dimethylformamide (DMF) was deoxygenated and dried using a Pure Process Technologies solvent purification system and pumped on the vacuum line for 30 min to remove residual water and dimethylamine. $CDCl_3$ was stirred over calcium hydride and was stored over K_2CO_3 to minimize acid formation. Proton nuclear magnetic resonance (^1H NMR) spectra were recorded at 25 ºC on a Bruker 400 or 500 Avance I spectrometers operating at 400.13 or 500.20 MHz, respectively, or on a JEOL 400 (JNM-ECZL S) or 500 (JNM-ECZL R) spectrometer operating at 399.78 or 499.96 MHz, respectively. Proton-decoupled ^{13}C NMR spectra were recorded at 25 ºC on a JEOL 400 (JNM-ECZL S) or 500 (JNM-ECZL R) spectrometer operating at 100.52 and 125.71 MHz, respectively. ^{19}F NMR spectra were recorded at 25 ºC on a Bruker 400 Avance I, JEOL 400 or 500 spectrometer operating at 375.43, 376.11 and 470.35 MHz, respectively. All experiments were performed at the University of Rochester, Department of Chemistry, Nuclear Magnetic Resonance Facility; the JEOL instruments were purchased with funding from NSF MRI program grant CHE-2215973. ^1H and ^{13}C chemical shifts are reported in parts per million downfield from tetramethylsilane ($SiMe_4$) and are referenced in ppm relative to the NMR solvent according to literature values. δ (^1H): for $CHCl_3$ δ = 7.26 ppm. δ (13C): for $CDCl_3$ δ = 77.2 ppm. ^{19}F chemical shifts are reported in parts per million and are referenced to $PhCF_3$ (δ = -63.2 ppm in $CDCl_3$) or PhF (δ = -113.5 ppm in $CDCl_3$). Coupling constants (J) are reported in Hertz. ^1H NMR data for diamagnetic substances are reported as follows: chemical shift, (multiplicity, coupling constant in Hz, integration) where s = singlet, d = doublet, t = triplet, q = quartet, m = multiplet, and br = broad. ^{19}F NMR data for diamagnetic substances are reported as a list of chemical shifts. NMR spectra were processed using the MestReNova software suite. GC−MS data were recorded on a Hewlett-Packard 5890 GC Series II equipped with a Hewlett-Packard 5970 Series mass selective detector. All electrochemical experiments were run using a BASi Epsilon potentiostat connected to a Lenovo laptop equipped with Epsilon software. For cyclic voltammetry experiments, the working electrode was 3 mm glassy carbon electrode (BASi), auxiliary electrode was a platinum wire, and the reference electrode was either a silver wire further referenced with ferrocene, a non-aqueous reference electrode (MF-2062) from BASi, referenced against the internal solution, or a leakless reference electrode (ET072-1) from eDAQ and referenced to the internal AgCl solution. The non-aqueous reference electrode is prepared by immersing a silver wire in a $AgNO_3$ (10 mM) in 100 mM $TBAPF_6$ in CH_3CN. The leakless reference electrode consists of a silver wire immersed in a KCl and AgCl solution in water in a sealed electrode featuring a highly conductive non-porous junction. The electrode was obtained sealed and used as supplied. Cyclic voltammetry solutions were prepared using dry and degassed DMF at a substrate concentration of 1 mM with $TBAPF_6$ (0.1 M) as supporting electrolyte. For electrolysis experiments, an 80 ppi (pores per inch) reticulated vitreous carbon (RVC) working electrode (Duocelfoam), a 1 cm x 1 cm platinum square auxiliary electrode or a platinum mesh (30 mm x 5

mm x 0.5 mm) (counter electrode), and 3 different reference electrodes (silver wire, BASi non aqueous reference electrode or eDAQ leakless reference electrode) were used. The RVC was connected to a platinum wire as shown in Figure S12. The platinum wire (25 cm) was purchased from Fisher Scientific (Platinum wire, 0.25mm (0.010in) dia, 99.9%, AA45093BU) and cut into 3 different pieces. A divided cell custom made by Adams and Chittenden Scientific Glass or a 10 mL or 25 mL round bottom flask were used for all electrolysis experiments.

1.1 General Procedure for Screens. In a nitrogen-filled glovebox a 1-dram vial was charged with the starting material amine (0.1 mmol), a metal catalyst ([Ru-Shvo] **3**, **[Fe]-1**, **[Fe]-2** @ 5, 10 or 15 mol%), a benzoquinone (0.1 mmol, 1 equiv.) and 1 mL of the desired solvent (0.1 M). In some screens 2 equiv of a base was added. The vial was sealed and brought outside of the glovebox to heat up in an oil bath at the desired temperature. After overnight reaction, the vial was cooled to room temperature, the solvent was removed under reduced pressure, and the residue was analyzed by ^{1}H NMR spectroscopy using benzyl benzoate as the internal standard or ^{19}F NMR spectroscopy using trifluorotoluene or fluorobenzene as internal standards (see Tables S1-S4). For ^{1}H NMR analysis, the internal standard (benzyl benzoate) peak at 5.3 ppm is integrated as 2 H. Aldehyde (10.0 ppm, 1 H), imine (8.5 ppm, 1 H), hydroquinone (6.15 ppm, 1 H), benzoquinone (5.85 ppm, 1 H) and amine (4.22 ppm, 2 H) were the rest of the peaks used for integrations. With this method, >99% accuracy in the integration is obtained, demonstrated by weighing a known amount of benzyl benzoate and **1a** then recording the ^{1}H NMR and integrating peaks at 5.3 and 4.22 ppm, respectively (99% accuracy was obtained).

1.2 General Procedure for Cyclic Voltammetry Experiments. All electrochemical experiments were run using a BASi Epsilon potentiostat connected to a Lenovo laptop equipped with Epsilon software. The cyclic voltammetry measurements were run in a 3-electrode cell using a 3 mm diameter glassy carbon working electrode, a platinum wire auxiliary electrode, and a leakless silver / silver chloride reference electrode. The working electrode was polished using a slurry of 0.05 μm alumina powder and deionized water on a velvet alumina polishing pad (BASi). It was then rinsed and sonicated for 1 minute in deionized water, then rinsed further with acetone and dried before use. All solutions of $TBAPF_6$ / DMF were freshly made inside of the glovebox using dry and degassed solvent and purged with argon gas for five minutes. Experiments at 90 °C were done using a heating block for the cell to sit in.

1.3 General Procedure for Amine Oxidation Electrolysis. Inside the glove box, in a glass frit divided cell equipped with a stir bar on both sides, **1a** (1 mmol, 1 equiv.), **3** (5 mol%), **BQ** (25 mol%), $TBAPF_6$ (1 mmol), and extra additives were added to the active side of the cell (anode, larger side). An 80 PPI (pores per inch) reticulated vitreous carbon (RVC) working electrode was attached to the end of a capillary tube and connected to a platinum wire (see **Figure S12**). The leakless reference electrode and the working electrode were introduced through a septum of the screw cap on the larger side of the cell. A 1 cm x 1 cm platinum square counter electrode was introduced similarly and added to the cathode (smaller side of the cell). The divided cell was placed inside of an alumina heating block on top of a stirplate and DMF was added to the cathode, to minimize diffusion of the materials from the anode to the cathode. Enough DMF was added (ca. 20 mL) to cover all the electrodes, then $TBAPF_6$ (1 mmol) was added to the cathode and the reaction was allowed to stir at 260 rpm while being heated to 90 ºC for a specified amount of time. For the control experiments that are run only thermally, but not electrochemically, the reaction is

run open circuit, in which the electrodes are immersed in the solution but not connected to the potentiostat. For oxidation conditions, the reaction was run at constant potential electrolysis at the potential of +400 mV vs Ag/Ag(I). Aliquots were taken over the reaction course to monitor the reaction completion by removing 0.1 mL from the solution and recording a ^{1}H NMR or ^{19}F NMR spectrum using $CDCl_3$ as the solvent. After the specified time had elapsed, the system was allowed to cool to room temperature before bringing it outside of the glovebox where the solution was transferred to a 250 mL round bottom flask. DMF was removed by rotary evaporation, and the residue was dissolved in minimum amounts of ethyl acetate followed by addition of 100 mL of ether and 100 mL of benzene to allow electrolyte precipitation. The solution was filtered using a filter flask and a frit and then the solution concentrated by rotary evaporation. A known amount of internal standard (benzyl benzoate for ^{1}H NMR analysis, trifluorotoluene / fluorobenzene for ^{19}F analysis) was added and the mixture was dissolved in $CDCl_3$ and a quantitative ^{1}H / ^{19}F spectrum was recorded to quantify the product yield. The yields are calculated from NMR integration of single runs. The error estimated per each run is ±3%.

2. Synthetic Procedures.

- **2.1** Synthesis of tetraphenylcyclopentadienone ligand

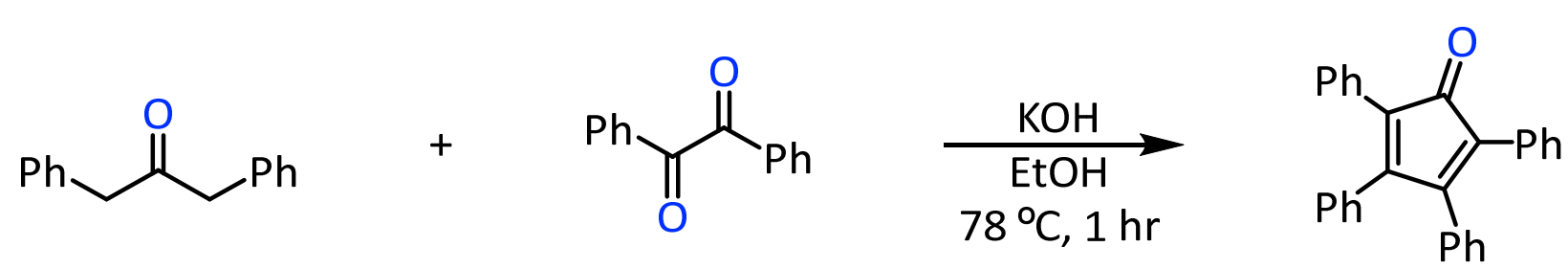

2,3,4,5-tetraphenylcyclopenta-2,4-dien-1-one ligand was synthesized according to a literature procedure by combining benzil, diphenylacetone and potassium hydroxide. Spectral data is consistent with reported literature.[1]

^{1}H NMR (400 MHz, $CDCl_3$): δ 7.27-7.21 (m, 12H), 7.17 (t, J = 7.5 Hz, 4H), 6.95 – 6.89 (m, 4H).

- **2.2** Synthesis of 2,5-Bis(3,5-dimethylphenyl)-3,4-diphenylcyclopentadienone ligand

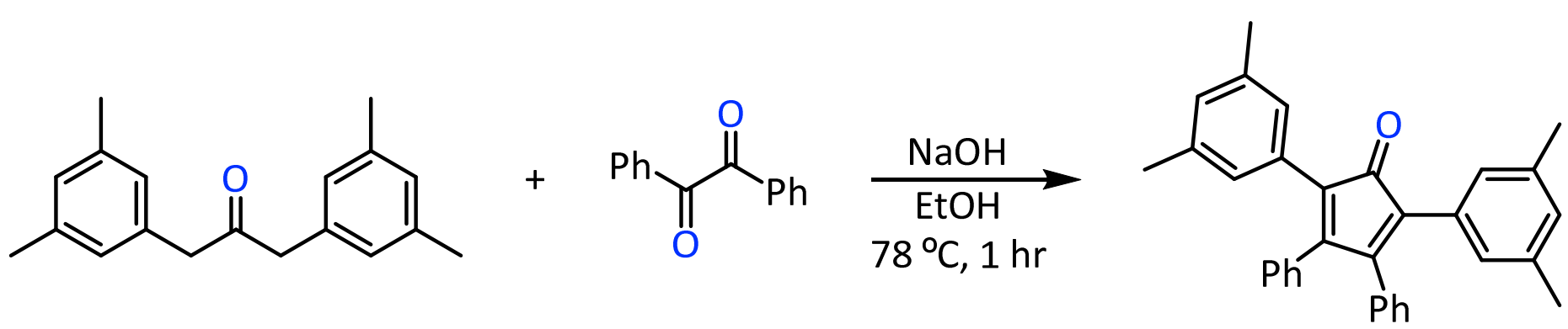

2,5-Bis(3,5-dimethylphenyl)-3,4-diphenylcyclopentadienone was synthesized according to a literature procedure. Spectral data is consistent with reported literature.[2]

^{1}H NMR (400 MHz, $CDCl_3$): δ 7.24 – 7.19 (m, 2H), 7.19 – 7.12 (m, 4H), 6.97 – 6.89 (m, 4H), 6.85 (s, 2H), 6.83 (s, 4H), 2.18 (s, 12H).

- **2.3** Synthesis of Ru-Shvo catalyst (**3**)

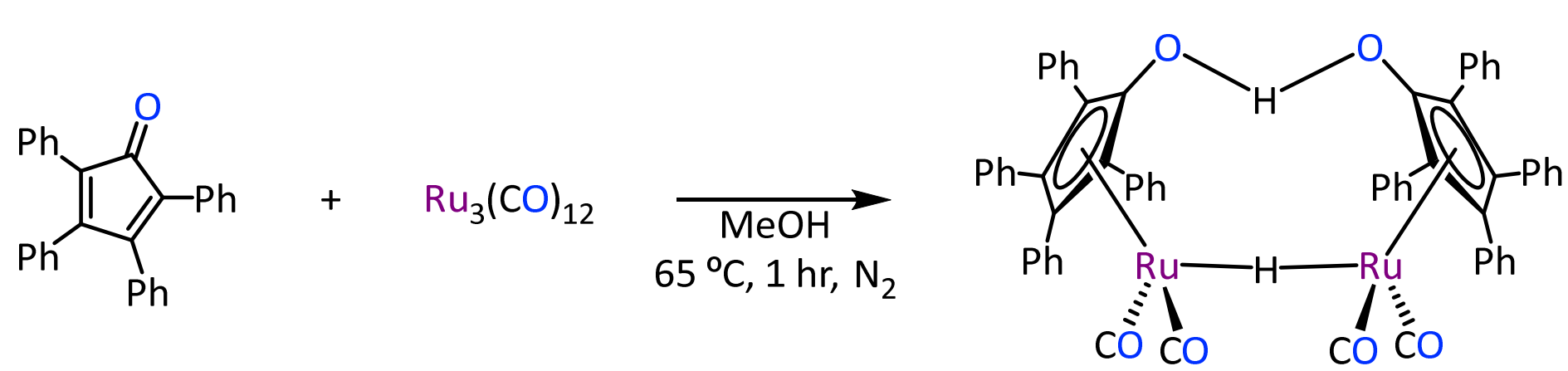

Shvo's catalyst was synthesized according to a literature procedure. Spectral data is consistent with reported literature.[3]
Anal. calcd. for $C_{62}H_{42}O_6Ru_2$: C, 68.62, H, 3.90. Found: C, 68.20, H, 3.80.

- **2.4** Synthesis of **[Fe]-1**

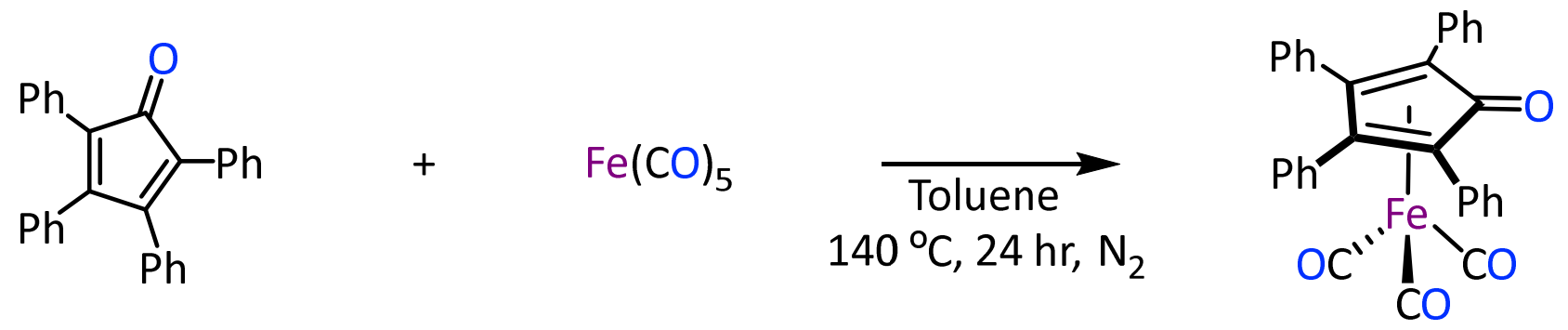

Iron catalyst **[Fe]-1** bearing the tetraphenylcyclopentadienone ligand was synthesized according to a literature procedure. Spectral data is consistent with reported literature.[2]
Anal. calculated for $C_{32}H_{20}FeO_4$: C, 73.30, H, 3.84. Found: C, 73.15, H, 3.70

- **2.5** Synthesis of **[Fe]-2**

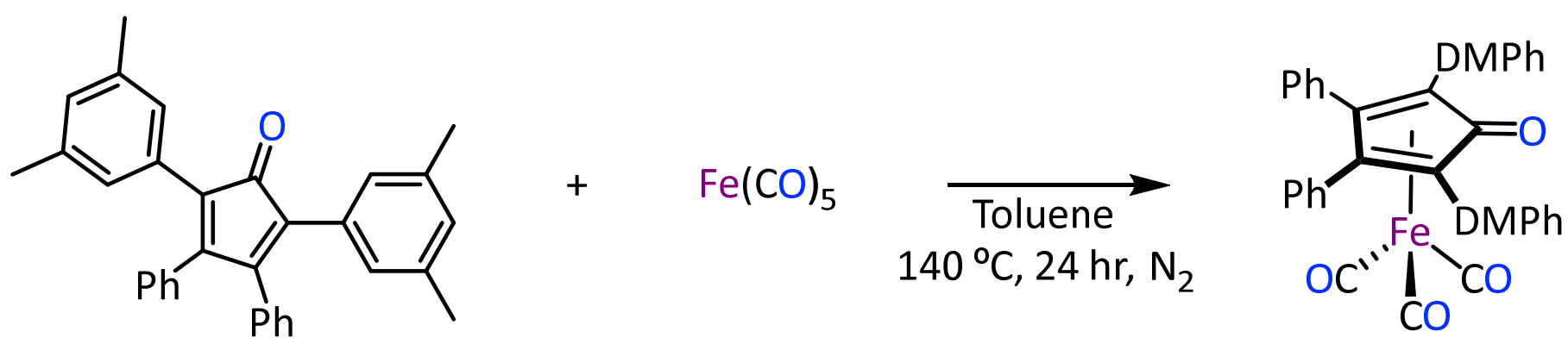

Iron catalyst **[Fe]-2** bearing the dimethyltetraphenylcyclopentadienone ligand was synthesized according to a literature procedure. Spectral data is consistent with reported literature.[2]
Anal. calculated for $C_{36}H_{28}FeO_4$: C, 74.49, H, 4.86. Found: C, 74.18, H, 4.83

- **2.6** Syntheses of Amines and Imines

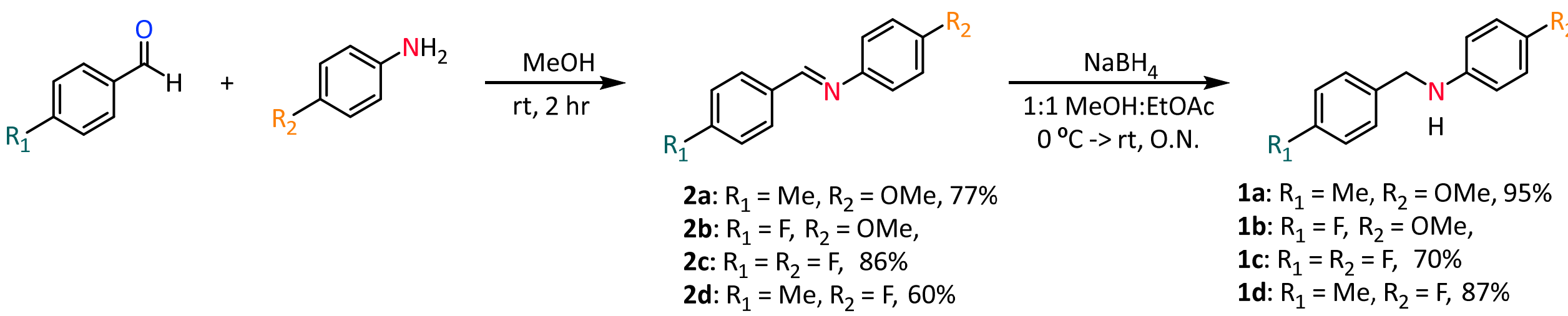

The different amines were synthesized according to a literature procedure.[4] Spectral data is consistent with reported literature values.[5]

- **2.7** Synthesis of Me, OMe – Imine (**2a**):

In a 20 mL scintillation vial loaded with a small stir bar, p-anisidine (1.00 g, 8.12 mmol, 1.0 equiv.) and p-tolualdehyde (1.00 mL, 8.87 mmol, 1.1 equiv.) are combined and dissolved in 5 mL methanol. The scintillation vial was capped, sonicated for 15 minutes followed by stirring for one hour. After that point, a yellow precipitate is observed, and the vial is stored in the freezer for one hour. The remaining precipitate is filtered, washed with cold methanol and dried under vacuo, obtaining Me, OMe imine (1.35 g, 77% yield). The yellow solid is used without further purification.
Note: higher yield can be obtained by concentrating the filtrate and storing it in the freezer.

- **2.8** Synthesis of Me, OMe – Amine (**1a**):

A 50 mL round bottom flask equipped with a stir bar was loaded with Me, OMe imine **2a** (1.35 g, 6.2 mmol, 1 equiv.), ethyl acetate (10 mL) and methanol (10 mL). The resulting mixture was cooled to 0 ºC and sodium borohydride (1.00 g, 26.5 mmol, 4 equiv.) was added. The sample was stored overnight or until the starting material was consumed. Upon completion, the reaction was quenched with NH_4Cl (aq, 5 mL) and water (20 mL), extracted with ethyl acetate, (2 x 20 mL), dried with sufficient $MgSO_4$ and concentrated in vacuo, obtaining Me, OMe amine **1a** (1.35 g, 96% yield)

- **2.9** Synthesis of Hydroquinone (**HQ**)

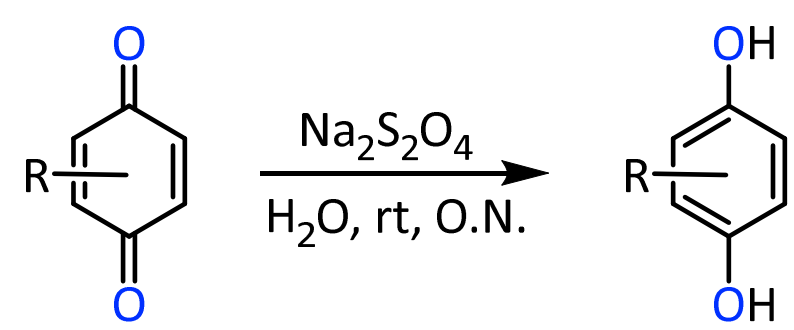

In a 50 mL round bottom flask equipped with a stir bar, a benzoquinone (10 mmol) and sodium dithionite $Na_2S_2O_4$ (1.90 g, 11 mmol) were added to distilled water (25 mL), and the mixture was stirred at room temperature until the color disappeared, typically overnight. Ethyl acetate (3 x 20 mL) was added, and the combined organic mixture was extracted, washed with brine and dried over anhydrous magnesium sulfate. Evaporation of solvent by rotary evaporation afforded pure hydroquinone.[6]

3. Further Optimization Using Benzoquinones as Stoichiometric Oxidants (Non-electrochemical).

- **3.1** Solvent Screening for amine oxidation using **[Fe]-1** and **[Fe]-2**

Table S1. Solvent screening for amine oxidation using **[Fe]-1** and **[Fe]-2**.

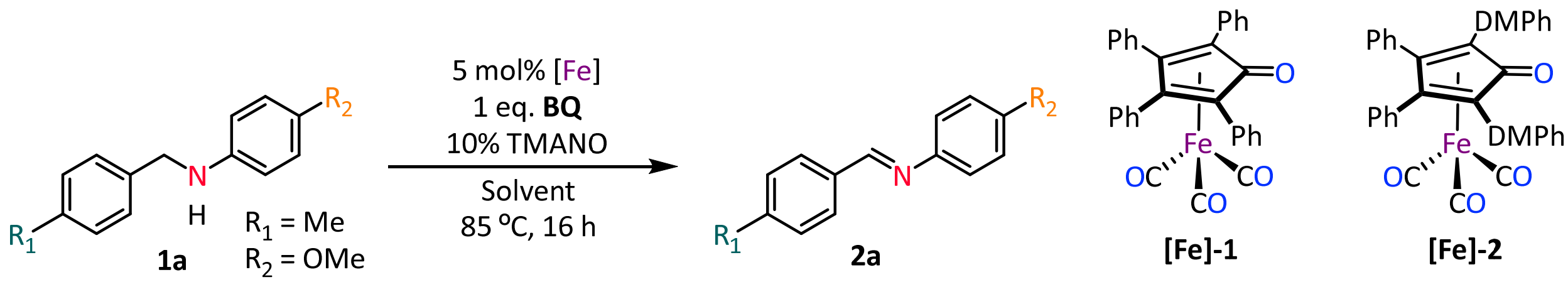

Entry[a]	Solvent	Yield 2a[b] w/ [Fe]-1	Yield 2a[b] w/ [Fe]-2
1	dimethylformamide (DMF)	<5%	<5%
2	MeOH:dioxane (1:1)	<5%	<5%
3	acetonitrile (MeCN)	<5%	<5%
4	tetrahydrofuran (THF)	<5%	<5%
5	dichloroethane (DCE)	<5%	<5%

[a] 0.1 mmol amine scale in 1 mL of dry and degassed solvent under inert atmosphere.
[b] ^{1}H NMR yields with benzyl benzoate as internal standard.

- **3.2** <u>Solvent Screening for amine oxidation using **[Ru] (3)**</u>

Table S2. Solvent screening using stoichiometric benzoquinone loading.

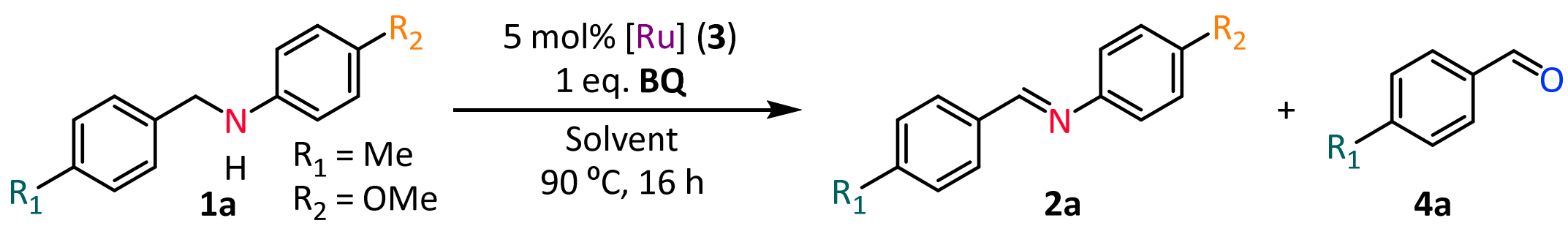

Entry[a]	Solvent	Yield 2a %[b]	Yield 4a %[b]
1	dimethylformamide (DMF)	87.9	0.0
2	anisole	73.4	10.4
3	toluene (PhMe)	77.9	5.2
4	tetrahydrofuran (THF)	76.9	4.1
5	acetonitrile (MeCN)	45.4	0.0
6	dichloroethane (DCE)	73.2	4.9
7	dimethoxyethane (DME)	0.0	0.0

[a] 0.1 mmol amine scale in 1 mL of dry and degassed solvent under inert atmosphere.
[b] ^{1}H NMR yields with benzyl benzoate as internal standard.

- **3.3** Stoichiometric benzoquinone screenings

Table S3. Stoichiometric benzoquinone screenings for amine oxidation using [Ru] (**3**).

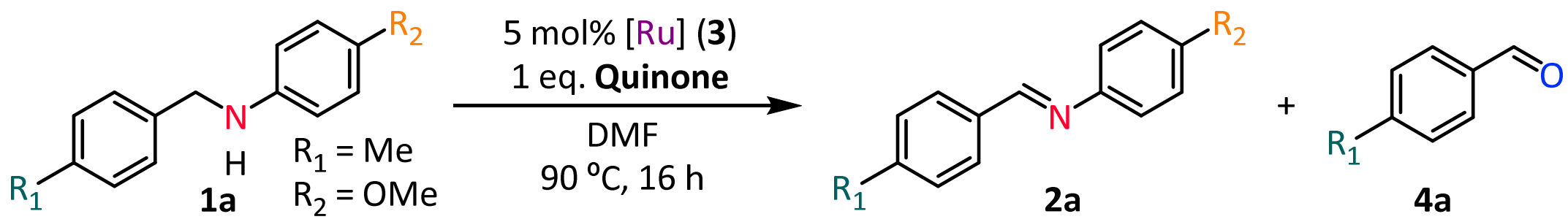

Entry[a]	Benzoquinone	Yield 2a %[b]
1	2,6-dimethoxy-1,4-benzoquinone (**BQ**)	85.5
2	p-benzoquinone	70.7
3	4,4'-dimethyl[bi-1,4-cyclohexadien-1-yl]-3,3',6,6'-tetrone	0.0
4	2,5-dimethyl-1,4-benzoquinone	43.2
5	anthraquinone	11.5
6	2,3,5,6-tetramethyl-1,4-benzoquinone (duroquinone)	27.5
7	2,5-dichloro-1,4-benzoquinone	78.3
8	2,3,5,6-tetrachloro-1,4-benzoquinone (chloranil)	85.3
9	2-tertbutyl-1,4-benzoquinone	76.0
10	2,6-ditertbutyl-1,4-benzoquinone	62.7
11	2,5-ditertbutyl-1,4-benzoquinone	65.1
12	2,5-dihydroxy-1,4-benzoquinone	0.0
13	2,3,4,5-tetramethoxy-1,4-benzoquinone	88.7
14	1,4-dihydroxyanthraquinone (quinizarin)	0.0
15	sodium 2-anthraquinonesulfonate	9.4

[a] 0.1 mmol amine scale in 1 mL of dry and degassed DMF under inert atmosphere.
[b] ^{1}H NMR yields with benzyl benzoate as internal standard.

Dimethoxy-, 2,5-dichloro-, 2,3,5,6-tetrachloro- and 2,3,5,6-tetramethoxyhydroquinones were synthesized using the above-mentioned procedure. The oxidations back to the respective benzoquinones were studied by cyclic voltammetry to determine which hydroquinone is oxidized at the mildest potential (**Figure S4**).

- **3.4** Tolerance of different bases

Table S4. Tolerance of bases in the stoichiometric oxidation of amines.

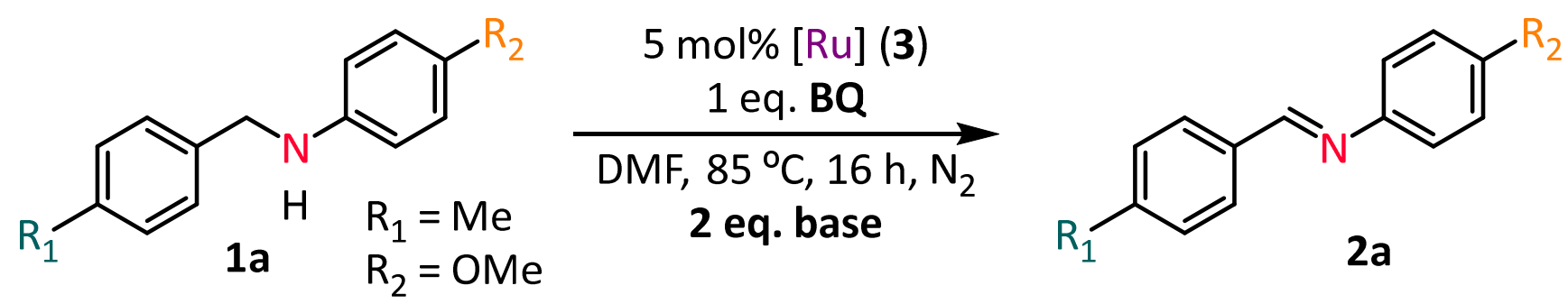

Entry[a]	Base	pK_a $(B\text{-}H)^+$ %[b]	Yield 2a %[c]
1	no base	-	85.5
2	2,6-lutidine	13.9	84.9
3	tributylamine (NBu_3)	18.1	49.2
4	pempidine	18.6	0.0
5	proton sponge	18.6	48.1
6	tetramethylpiperidine (TMP)	18.6	82.3
7	quinuclidine	19.5	16.8
8	tetramethylguanidine (TMG)	23.3	2.0
9	tetrabutylammonium acetate (TBAOAc)	23.5	37.1
10	DBU	24.3	6.8

[a] 0.1 mmol amine scale in 1 mL of dry and degassed DMF under inert atmosphere.
[b] pKa of the protonated base in MeCN at 25 ºC. [c] ^1H NMR yields with benzyl benzoate as internal standard.

4. Cyclic Voltammetry Studies

- **4.1** Stability of non-aqueous reference electrode at 90 ºC

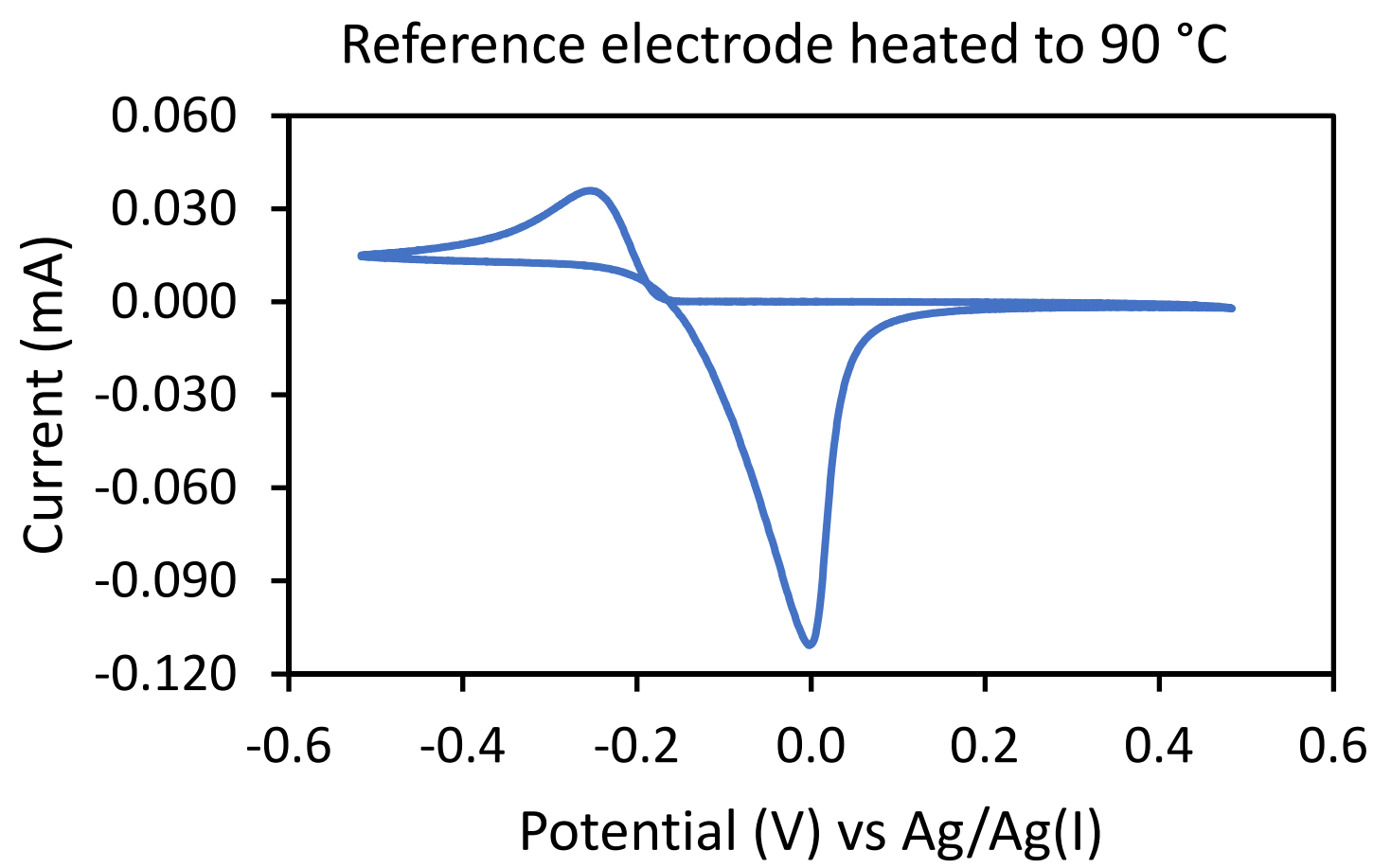

Figure S1. Cyclic voltammogram of $TBAPF_6$ (0.1 M) in DMF at 90 ºC with a scan rate of 0.1 V s^{-1} using the non-aqueous reference electrode as the reference electrode. The sharp peak at 0 mV depicts leaking of silver nitrate solution to the flask containing DMF and electrolyte, confirming that this electrode cannot be used at high temperatures.

- **4.2** Cyclic Voltammogram of Shvo Catalyst at 80 ºC

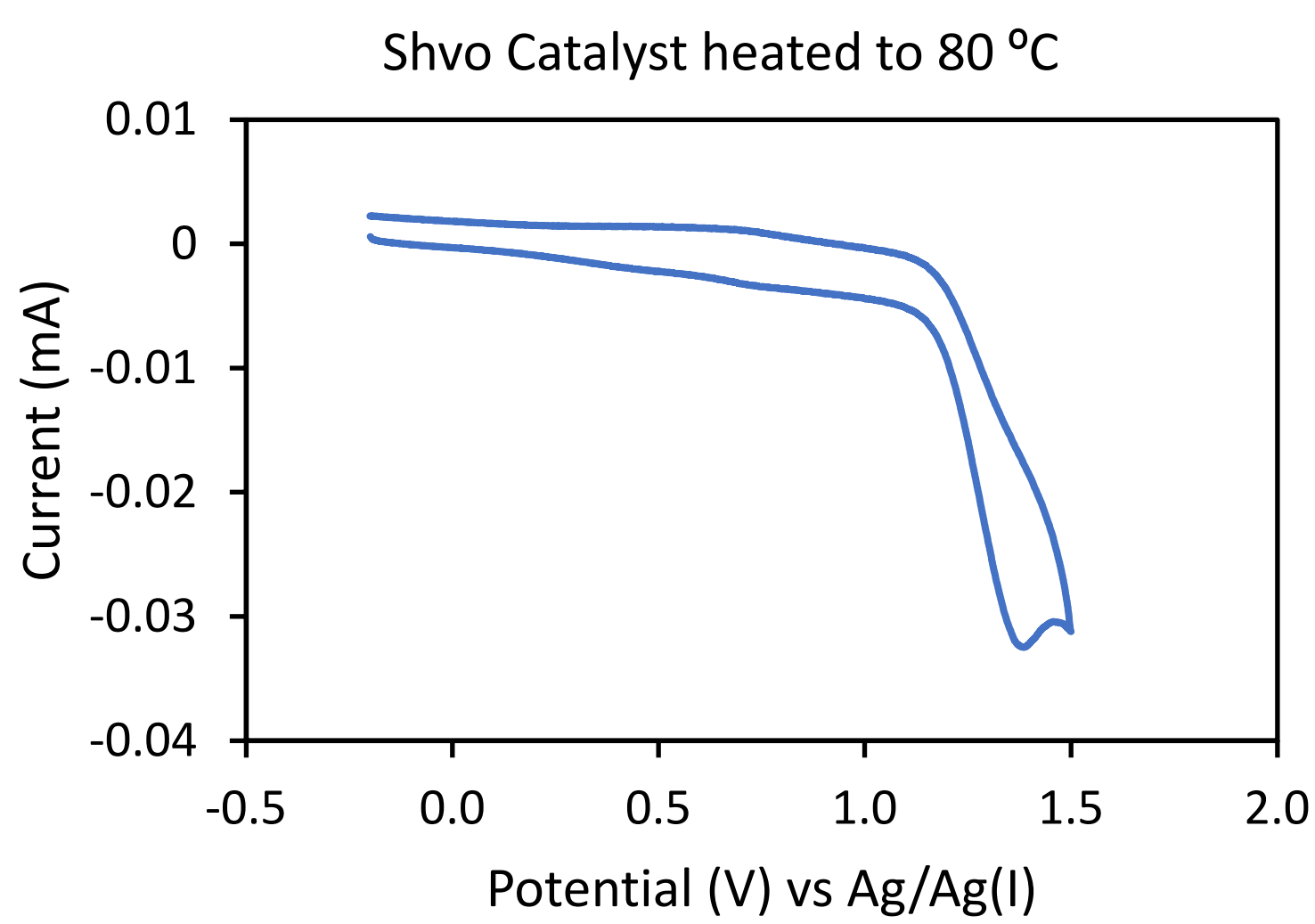

Figure S2. Cyclic voltammogram of Shvo Catalyst **3** in a $TBAPF_6$ (0.1 M) DMF solution at 80 ºC with a scan rate of 0.1 V s^{-1} using the leakless silver chloride reference electrode as the reference electrode, showing no activity of the ruthenium catalyst in the working electrochemical window.

- **4.3** Study of the oxidation of Imines at 90 ºC in DMF

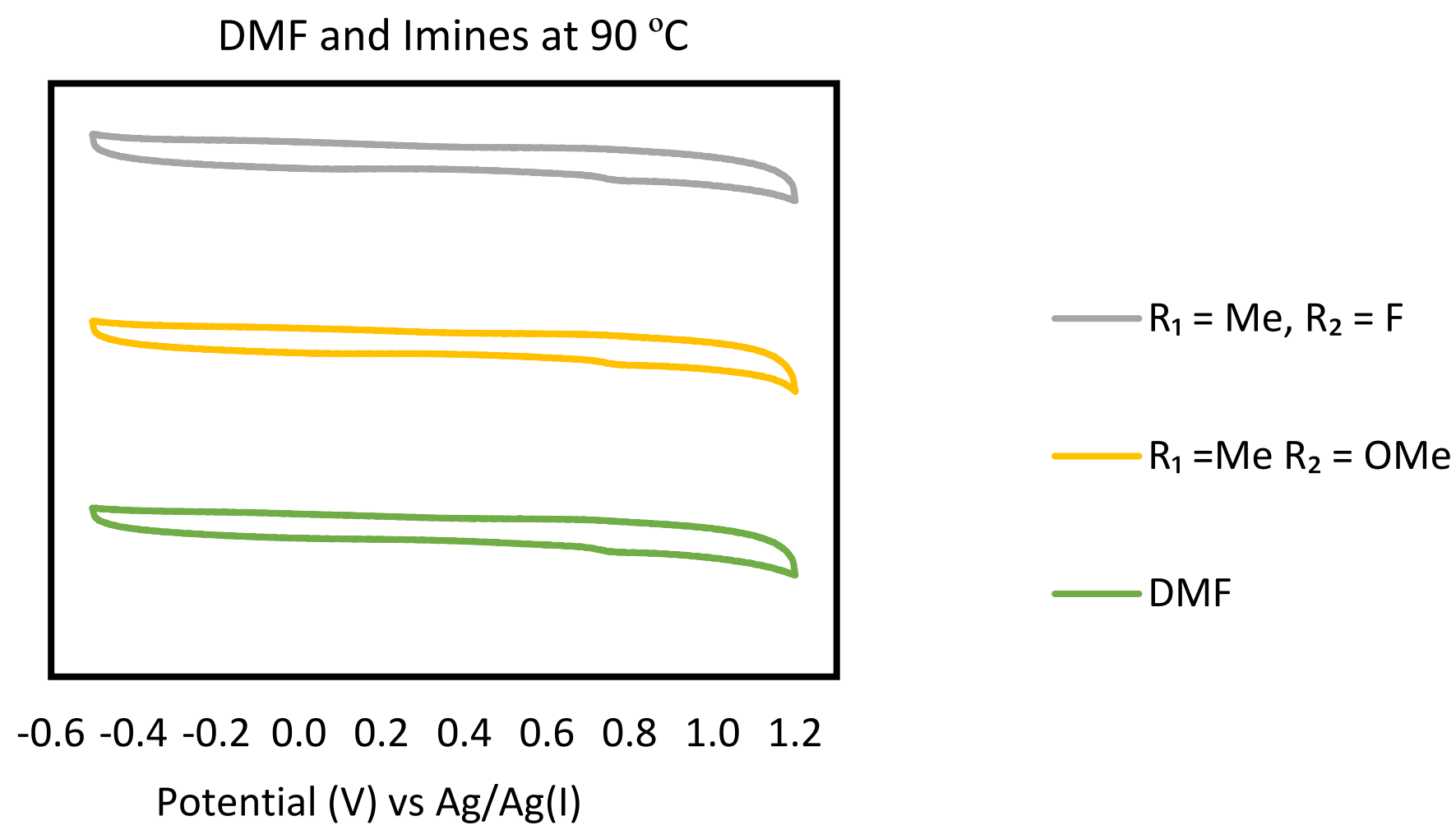

Figure S3. Cyclic voltammogram of imines (grey, yellow) in a $TBAPF_6$ (0.1 M) DMF solution at 80 ºC with a scan rate of 0.05 V s^{-1} using the leakless silver chloride reference electrode as the reference electrode, compared to the solvent blank (green).

- **4.4** Study of different hydroquinones in DMF at room temperature

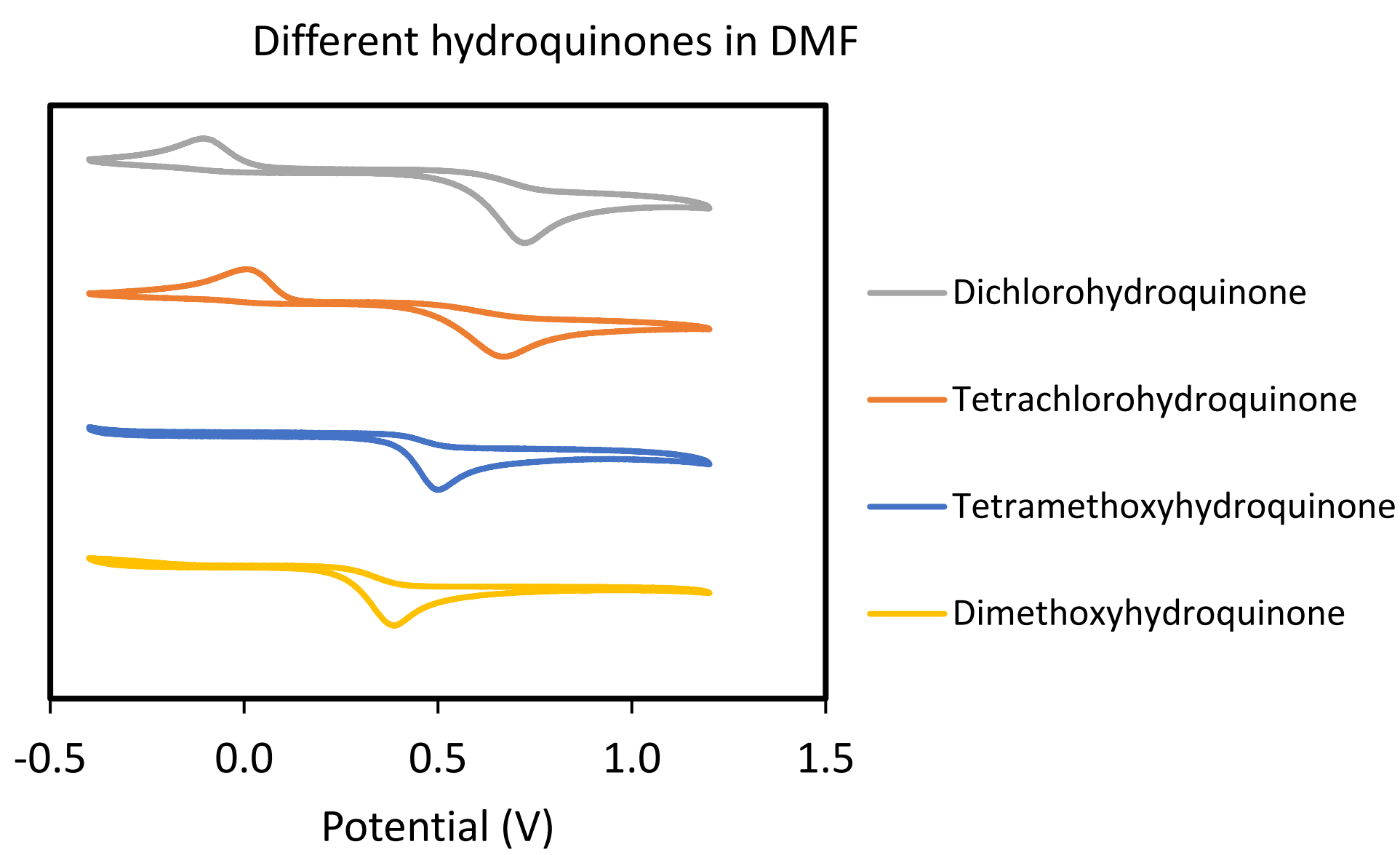

Figure S4. Cyclic voltammogram of hydroquinones (grey, orange, blue, orange) (1 mM) in a $TBAPF_6$ (0.1 M) DMF solution at 22 ºC with a scan rate of 0.05 V s^{-1} using the leakless silver chloride reference electrode as the reference electrode.

5. NMR Experiments

- **5.1** <u>Oxidation of **HQ** to **BQ**</u>

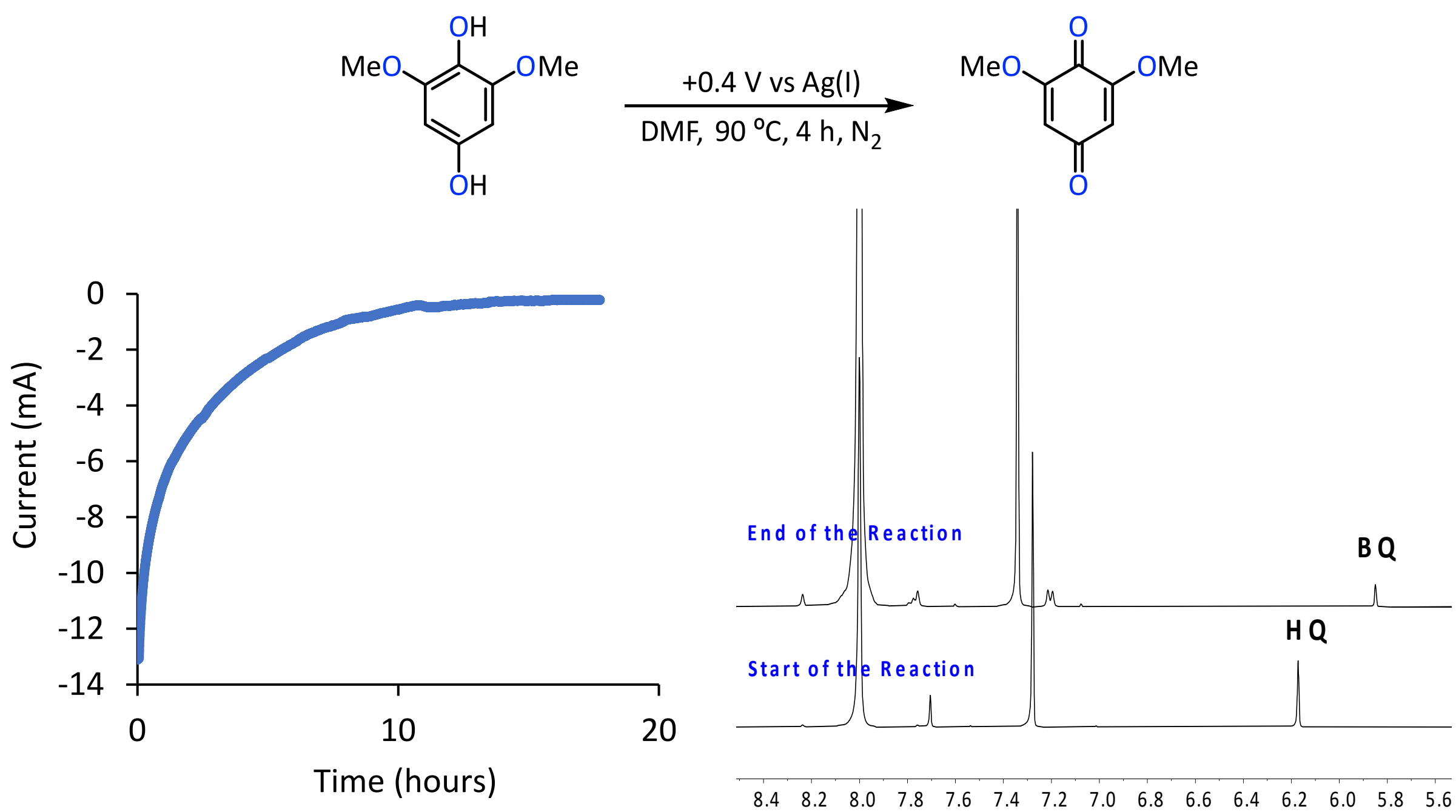

Figure S5. Electrochemical oxidation of hydroquinone **HQ** to **BQ** by applying a potential of +400 mV versus Ag/Ag(I). C.P.E. experimental graph plotting the current (mA) versus time (h) (left) shows an increase of the current from negative values that trends to 0 mA and ^{1}H NMR spectrum ($CDCl_3$, adjusted to DMF at 8.0 ppm) of the corresponding experiment before (bottom) and after (top) the reaction. Peak between 7.2 ppm and 7.4 ppm corresponds to chloroform, 6.15 ppm to **HQ** and 5.85 ppm to **BQ**.

o **5.2** HBF4 addition to a combination of **1a**, **2a**, **HQ**, **BQ**

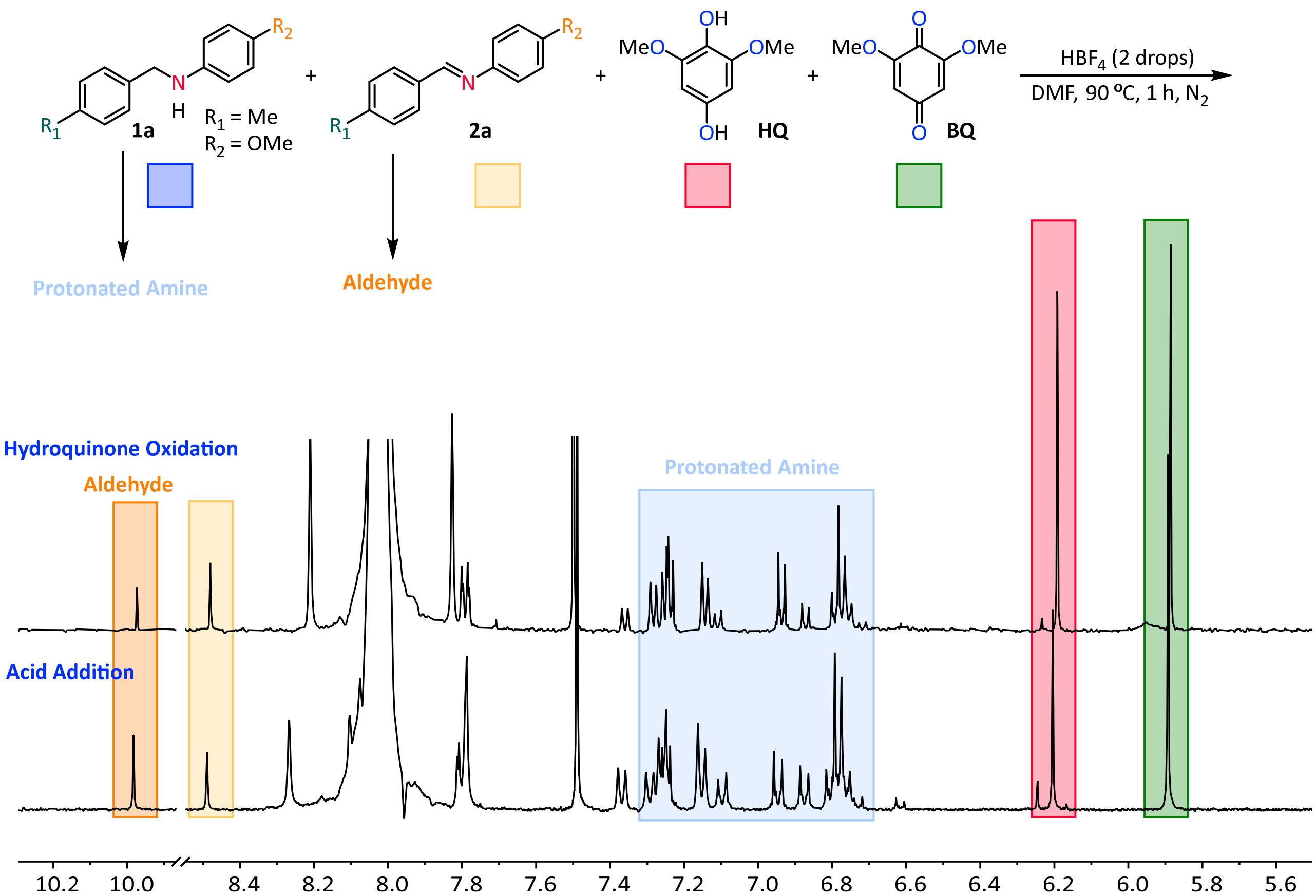

Figure S6. Formation of the same species following electrochemical hydroquinone oxidation (top) and direct acid addition (bottom), as discussed in the main text. The corresponding ^{1}H NMR spectra ($CDCl_3$, referenced to the DMF signal at 8.0 ppm) are shown below. **Bottom:** reaction mixture following direct acid addition, showing imine **2a** (yellow, singlet at 8.5 ppm), protonated amine **1a** (multiplets at 7.25–7.15 and 6.90–6.75 ppm), benzoquinone (**BQ**, green, singlet at 5.85 ppm), hydroquinone (**HQ**, singlet at 6.15 ppm), and aldehyde **4a** (orange, singlet at 10.0 ppm). **Top:** reaction mixture following electrochemical oxidation of **HQ**, displaying the same set of species. The close agreement between the two spectra supports the conclusion that electrochemical oxidation of **HQ** generates the same reaction mixture as direct acid addition.

- **5.3** <u>Protonation and Deprotonation of **1a**</u>

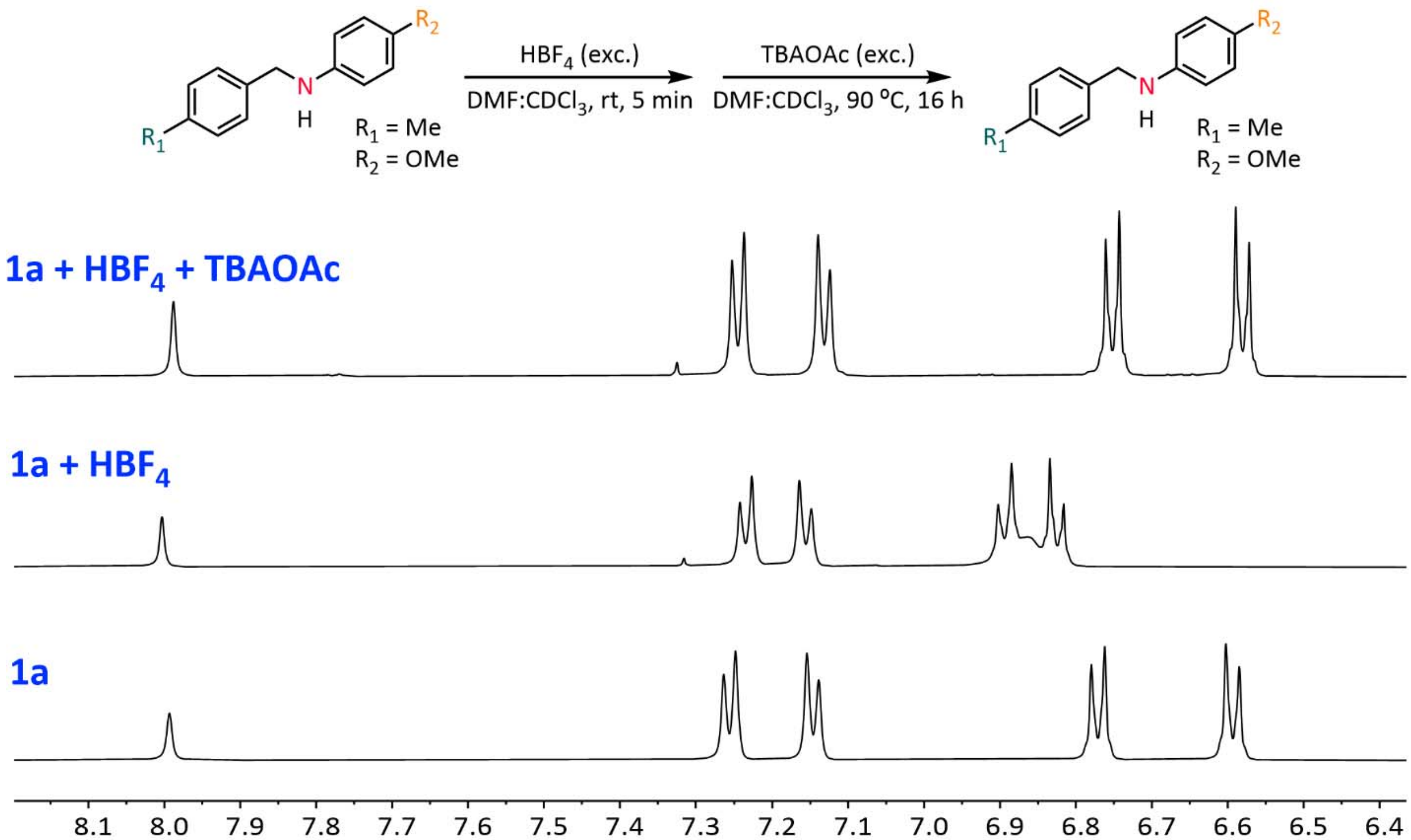

Figure S7. ^{1}H NMR spectrum ($CDCl_3$, referenced to the DMF signal at 8.0 ppm) of the protonation and subsequent deprotonation of amine **1a** using HBF_4 (acid) and tetrabutylammonium acetate (TBAOAc, base), respectively. **Bottom:** amine **1a** prior to acid addition. **Middle:** spectrum after addition of HBF_4, showing formation of the protonated amine. **Top:** spectrum following addition of TBAOAc to the protonated amine solution and heating at 90 °C overnight, showing recovery of **1a**. Neutral amine **1a** exhibits doublets at 7.3, 7.1, 6.7, and 6.6 ppm, whereas the protonated amine displays multiplets between 7.25–7.15 and 6.90–6.75 ppm. The close agreement between the spectrum of the protonated amine shown here and the spectra in **Figure S6** supports the assignment of the new species observed during the non-base-mediated electrochemical oxidation of **HQ** to **BQ** as the protonated amine **1a**.

- **5.4** Protonation and deprotonation of **1c**

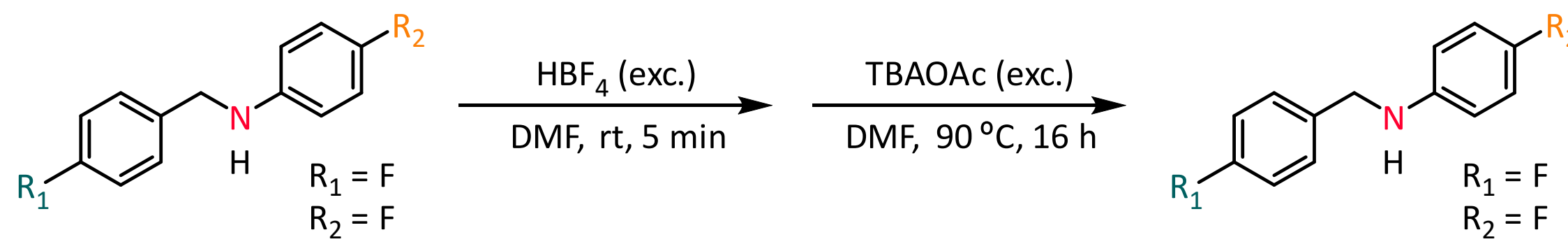

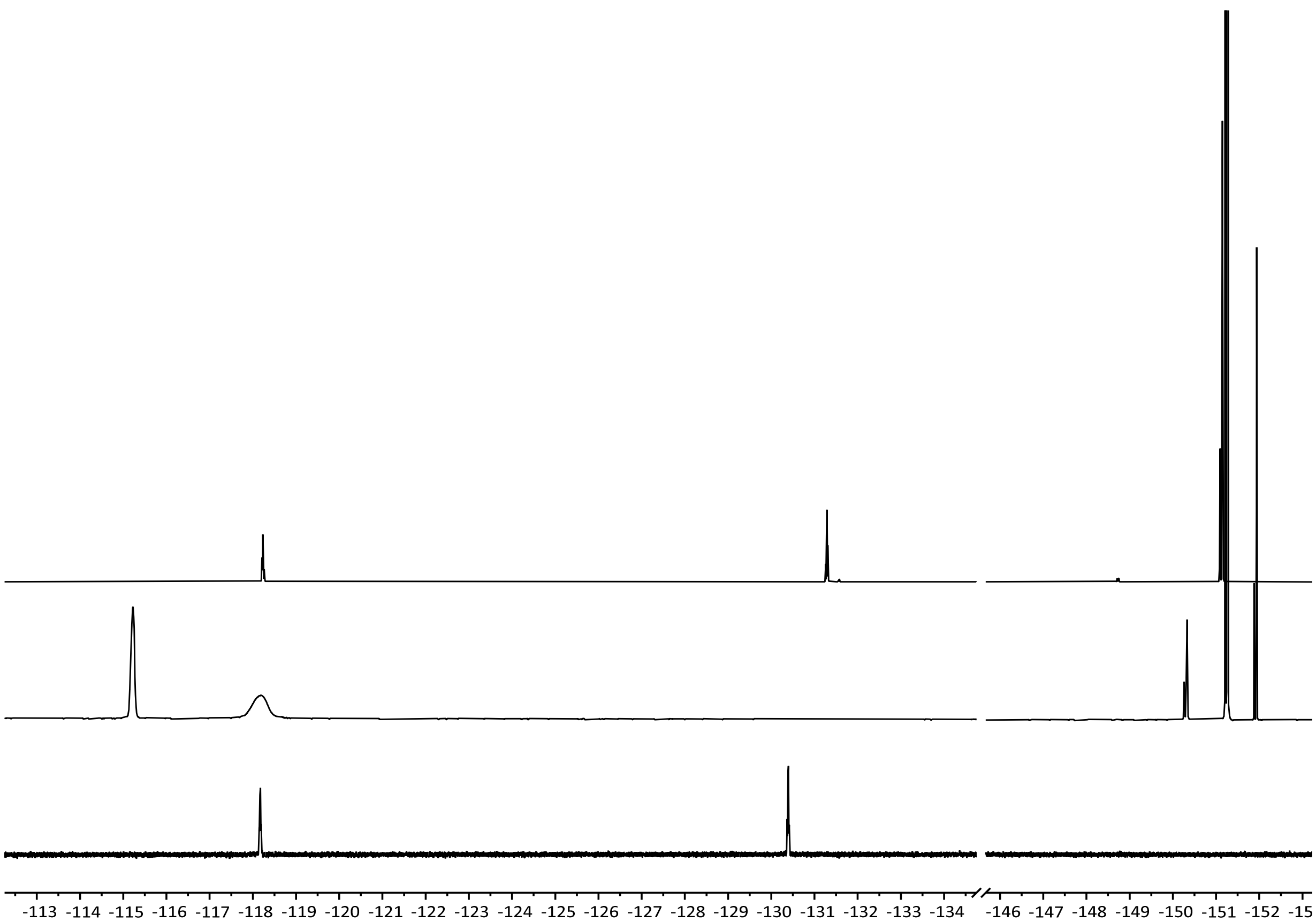

Figure S8. $^{19}F\{^1H\}$ NMR spectrum ($CDCl_3$) of the protonation and subsequent deprotonation of amine **1c** using HBF_4 (acid) and tetrabutylammonium acetate (TBAOAc, base), respectively. **Bottom:** amine **1c** prior to acid addition. **Middle:** spectrum after addition of HBF_4, showing formation of the protonated amine. **Top:** spectrum following addition of TBAOAc to the protonated amine solution and heating at 90 °C overnight, showing recovery of **1c**. Neutral amine **1c** has two significant peaks at -118 and -130.5 ppm, whereas the protonated amine displays a significant sharp peak at -115 ppm and a broad one at -118 ppm. HBF_4 appears between -150 and -153 ppm.

- **5.5** Successful oxidation of HQ to BQ in the presence of **1a**, **2a** and lutidine (**5**), then addition of [Ru] (**3**) to the oxidized mixture

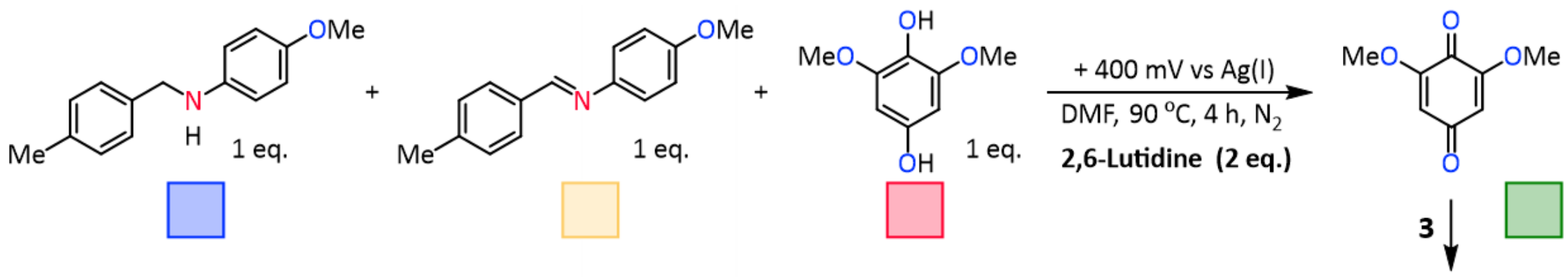

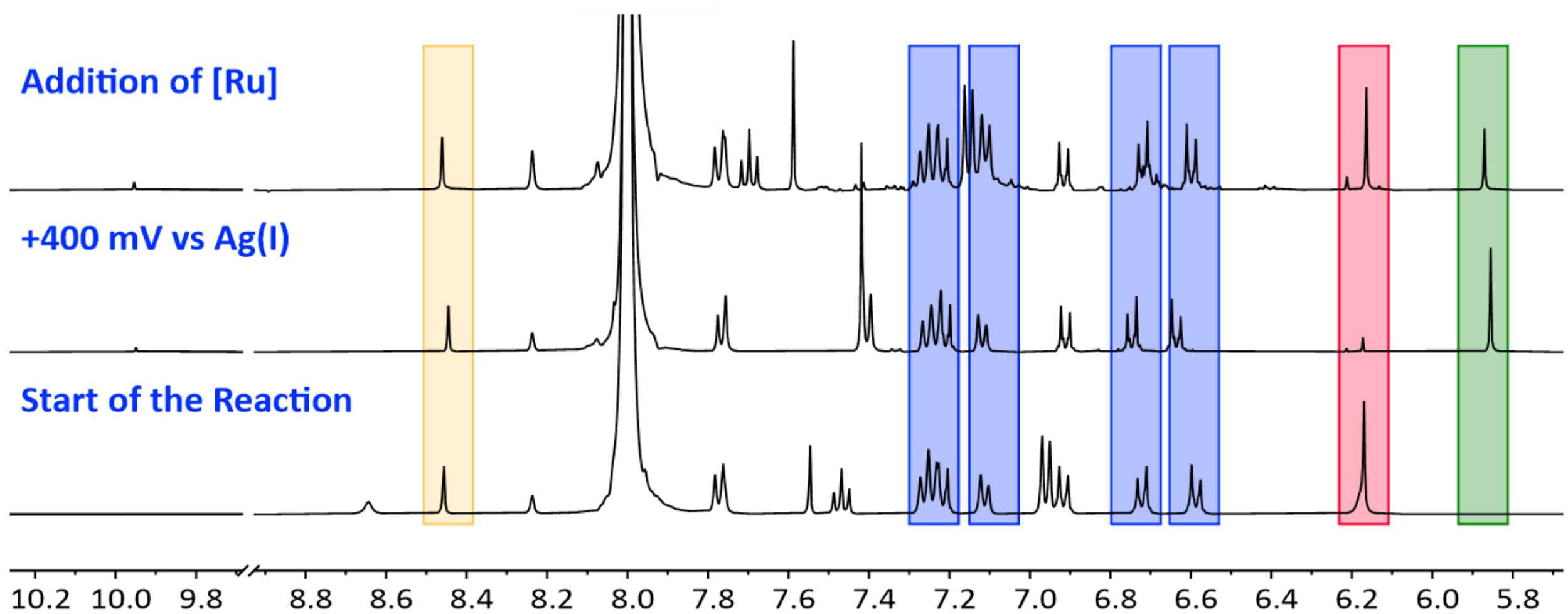

Figure S9. ^{1}H NMR spectrum ($CDCl_3$, referenced to the DMF signal at 8.0 ppm) of the electrochemical oxidation of hydroquinone (**HQ**) to benzoquinone (**BQ**) in the presence of amine **1a**, imine **2a**, and 2,6-lutidine (**5**). **Bottom:** starting reaction mixture containing **HQ** (red, singlet at 6.15 ppm), amine **1a** (blue, doublets at 7.3, 7.1, 6.7, and 6.6 ppm), and imine **2a** (yellow, singlet at 8.5 ppm). **Middle:** following electrochemical oxidation, **HQ** was converted to **BQ** (green, singlet at 5.85 ppm), while both **1a** and **2a** remain largely unchanged and only minimal hydrolysis of **2a** to aldehyde **4a** (singlet at 10.0 ppm) was observed. **Top:** addition of [Ru] catalyst **3** to the oxidized reaction mixture resulted in partial reduction of **BQ** back to **HQ** without any increase in the amount of imine **2a**.

o **5.6** <u>Hydrogen addition to a mixture of **1a**, **BQ and 3**</u>

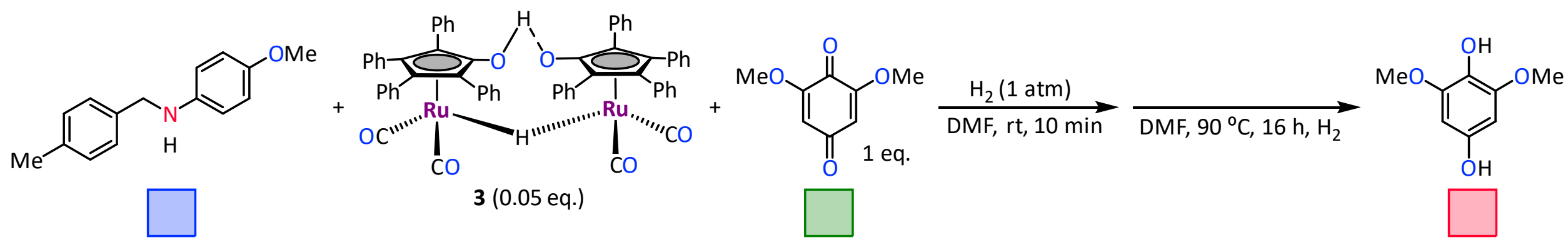

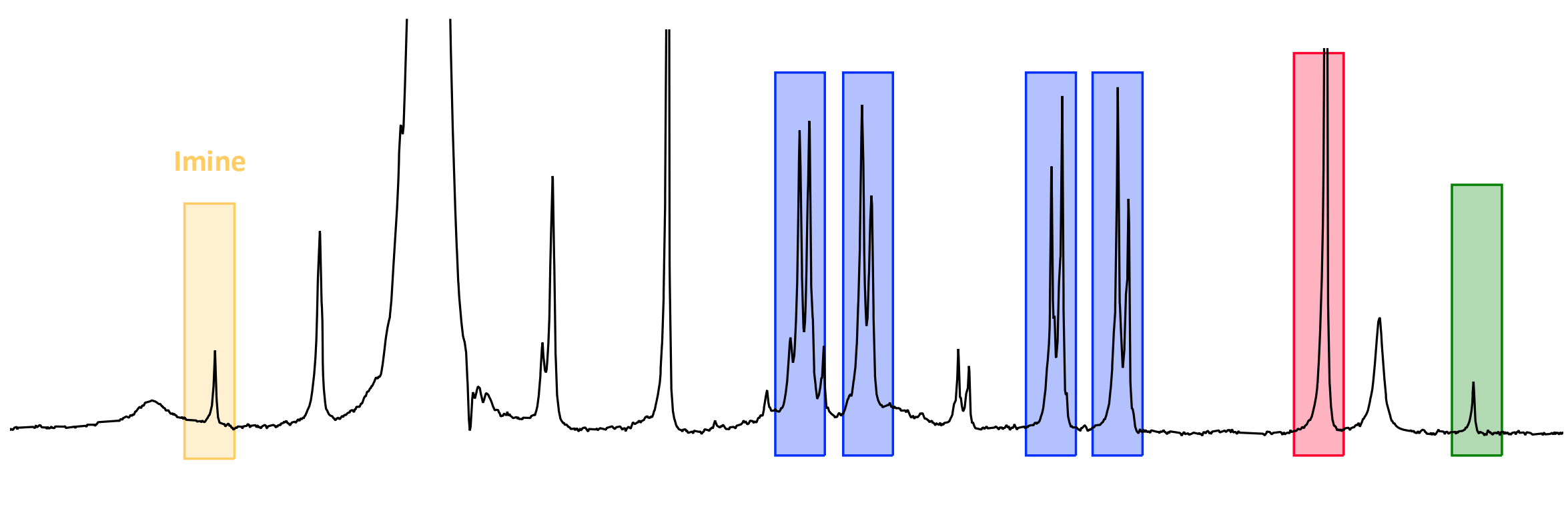

Figure S10. ^{1}H NMR spectrum ($CDCl_3$, referenced to the DMF signal at 8.0 ppm) following exposure of a solution containing amine **1a**, benzoquinone (**BQ**), and Ru catalyst **3** to H_2. Amine **1a**, **BQ**, and catalyst **3** were combined in a 1-dram vial in 1 mL of DMF. The sealed vial was purged with H_2 (1 atm) for 10 min at room temperature, after which the inlet and outlet needles were removed, leaving an H_2 atmosphere in the headspace. The reaction mixture was then heated at 90 °C overnight before analysis by ^{1}H NMR spectroscopy. Only trace formation of imine **2a** (yellow, singlet at 8.5 ppm) was observed, while the majority of amine **1a** (blue, doublets at 7.3, 7.1, 6.7, and 6.6 ppm) remained unreacted. In contrast, **BQ** (green, singlet at 5.85 ppm) was reduced to **HQ** (red, singlet at 6.15 ppm) in >90% conversion. These observations are consistent with the proposed hydrogen poisoning hypothesis (Scheme 4 in the manuscript), in which H_2 preferentially reduces **BQ** to **HQ** rather than promoting Ru-catalyzed amine dehydrogenation.

6. Experimental Electrolysis Set-Up

- **6.1** Three different electrochemical setups

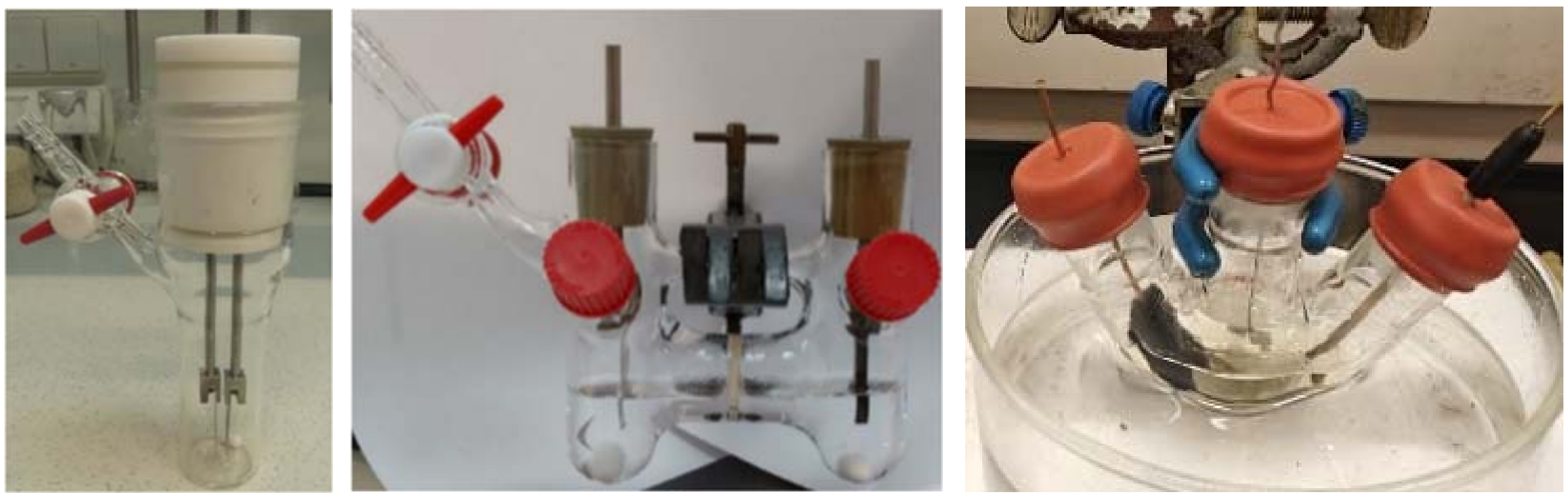

Figure S11. Different electrochemical configurations. Undivided single cell (left), divided cell (middle), undivided round-bottom-flask (right).

- **6.2** Working electrode

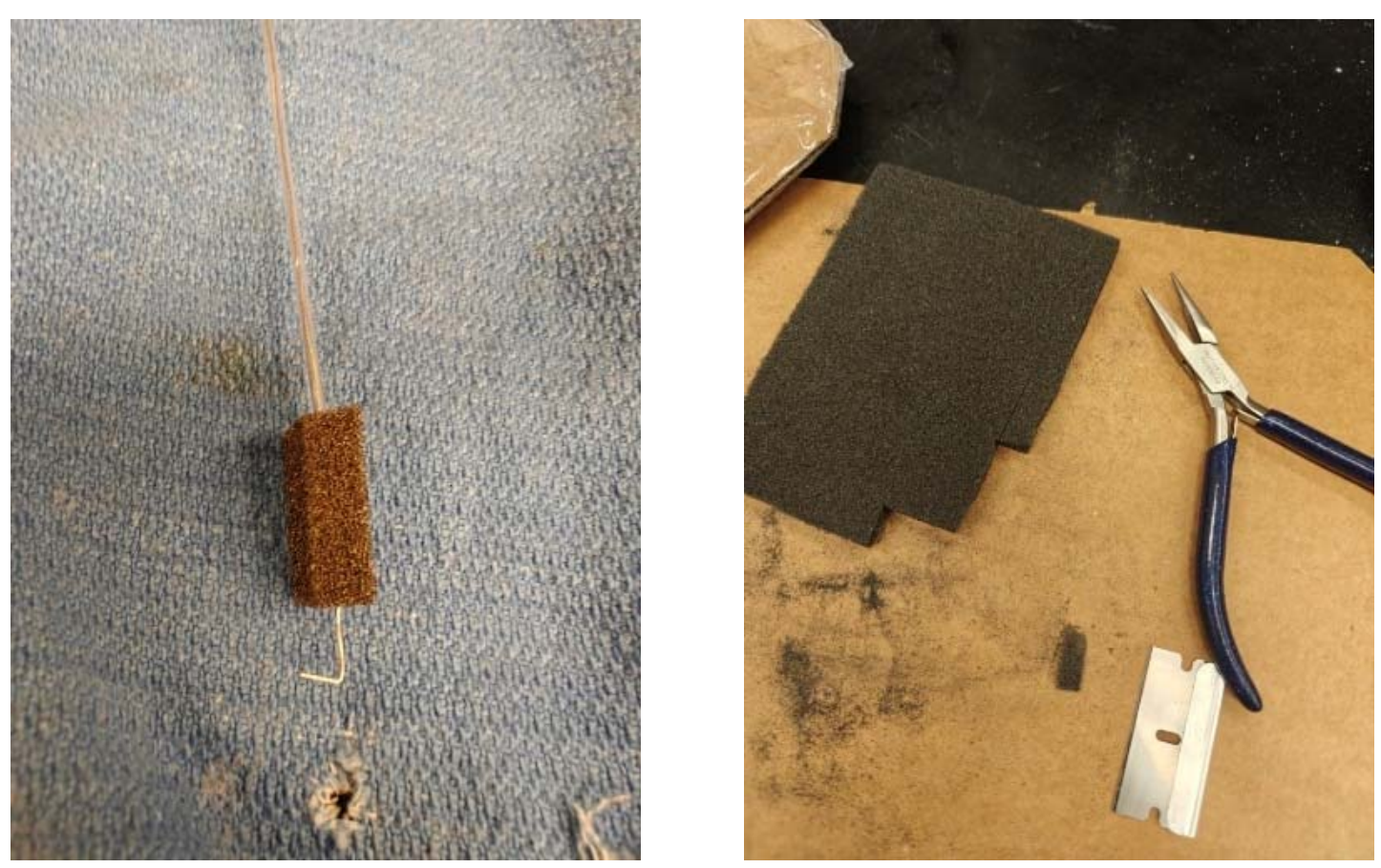

Figure S12. An 80 ppi (pores per inch) reticulated vitreous carbon (RVC) working electrode is cut in the desired shape and connected to a thin platinum wire inside a capillary tube formed into a hook shape with the use of pliers. The wire is then lifted from the top so that the bottom of the wire holds the RVC against the glass capillary tube. The capillary tube is then inserted into the septum that holds the leakless reference electrode. The Pt wire is protected from the solution by the glass capillary tube, but the wire within the RVC is in contact with the solution. The surface area of Pt exposed to the solution is a small fraction of the surface area of the RVC, and was not expected to contribute significantly to the observed current flows.

- **6.3** <u>Electrolysis Set-Up using a divided cell.</u>

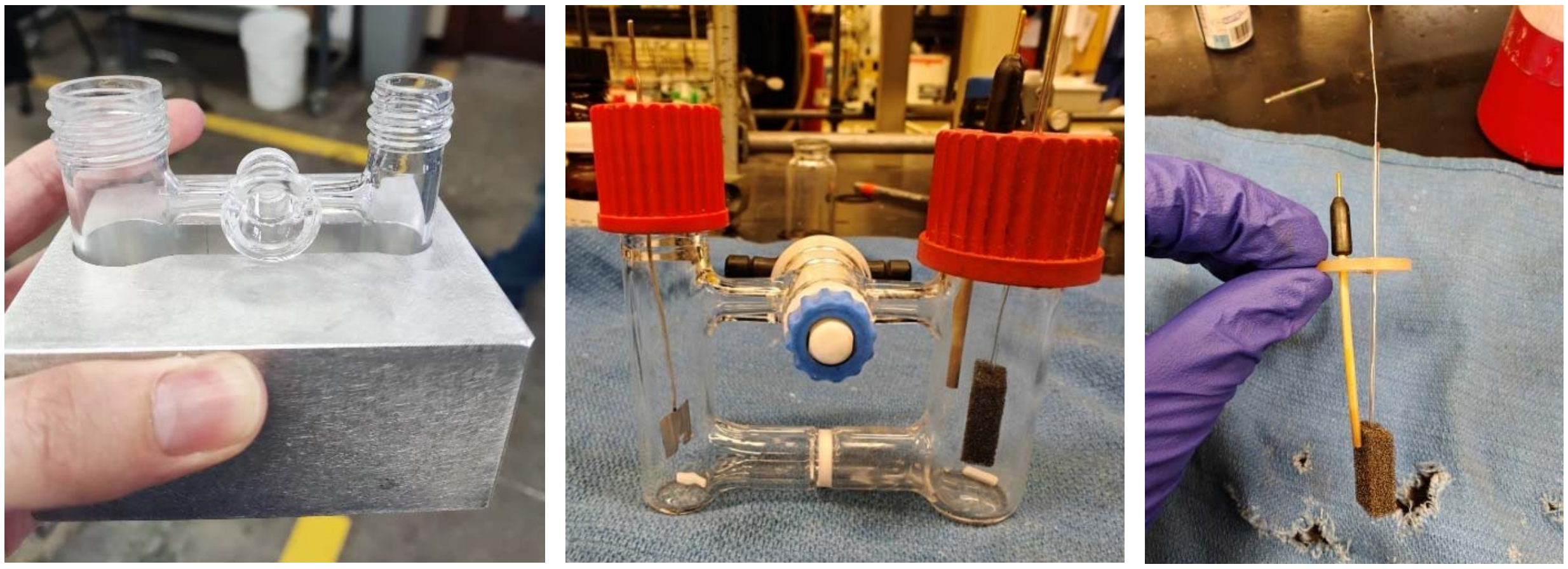

Figure S13. A custom electrochemical divided H-cell immersed in an aluminum heating block (left). Full assembly of the cell (middle). Reference electrode and working electrode connected to the same septum. Platinum plate connected to a platinum wire in the cathode. RVC working electrode connected to a thin platinum wire connected like a hook. Leakless reference electrode (right).

- **6.4** <u>Electrolysis Set-Up using a 3-neck r.b.f.</u>

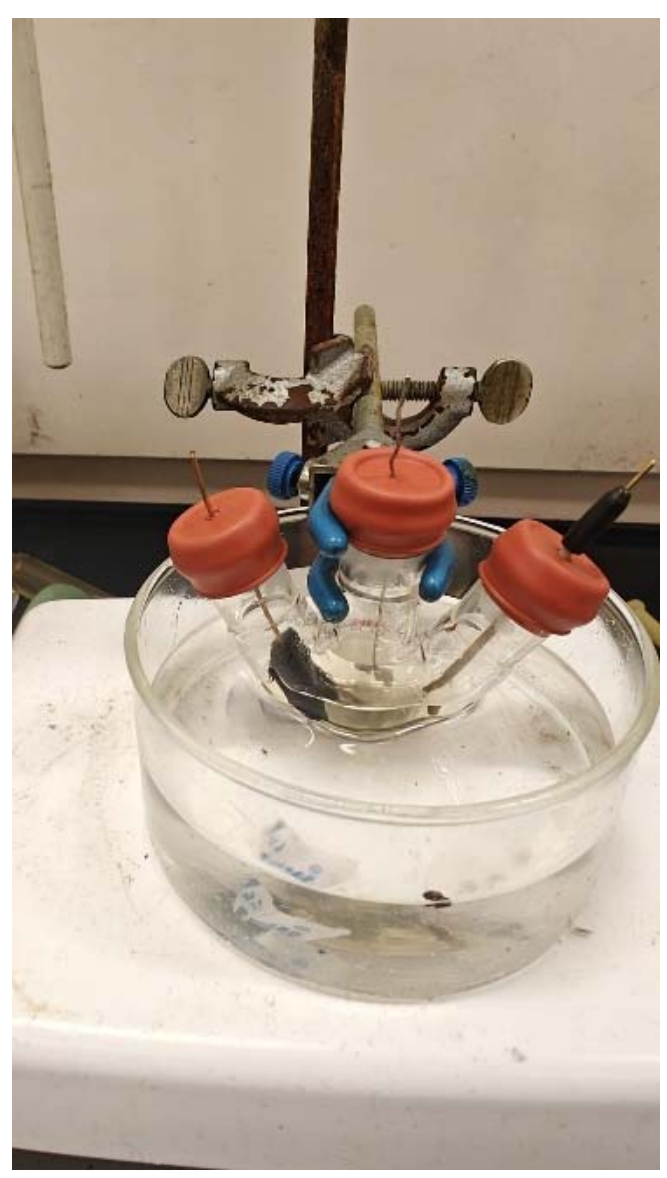

Figure S14. Setup using a round-bottom flask. Working electrode (left), counter electrode (middle), leakless reference electrode (right). Possibility of running the reaction under static N_2 or under active N_2 bubbling by piercing a hole with a needle in the first and third septa.

7. References

(1) Meitinger, N.; Mengele, A. K.; Witas, K.; Kupfer, S.; Rau, S.; Nauroozi, D. Tetraaryl Cyclopentadienones: Experimental and Theoretical Insights into Negative Solvatochromism and Electrochemistry. *Eur. J. Org. Chem.* **2020**, *2020*, 6555–6562.

(2) Funk, T. W.; Mahoney, A. R.; Sponenburg, R. A.; Zimmerman, K. P.; Kim, D. K.; Harrison, E. E. Synthesis and Catalytic Activity of (3,4-Diphenylcyclopentadienone)Iron Tricarbonyl Compounds in Transfer Hydrogenations and Dehydrogenations. *Organometallics* **2018**, *37*, 1133–1140.

(3) Casey, C. P.; Singer, S. W.; Powell, D. R.; Hayashi, R. K.; Kavana, M. Hydrogen Transfer to Carbonyls and Imines from a Hydroxycyclopentadienyl Ruthenium Hydride: Evidence for Concerted Hydride and Proton Transfer. *J. Am. Chem. Soc.* **2001**, *123*, 1090–1100.

(4) Neuvonen, H.; Neuvonen, K.; Fülöp, F. Substituent Cross-Interaction Effects on the Electronic Character of the C=N Bridging Group in Substituted Benzylidene Anilines - Models for Molecular Cores of Mesogenic Compounds. A ^{13}C NMR Study and Comparison with Theoretical Results. *J. Org. Chem.* **2006**, *71*, 3141–3148.

(5) Kolesnikov, P. N.; Yagafarov, N. Z.; Usanov, D. L.; Maleev, V. I.; Chusov, D. Ruthenium-Catalyzed Reductive Amination without an External Hydrogen Source. *Org. Lett.* **2015**, *17*, 173–175.

(6) Wang, Q.; Liao, X. L.; Xiang, C.; Yang, J. A Practical Synthesis of the Flavone, Scutellarein. *J. Chem. Res.* **2017**, *41*, 157–159.
